# Therapeutic Effect of *Padina arborescens* Extract on a Cell System Model for Parkinson’s Disease

**DOI:** 10.3390/neurosci5030024

**Published:** 2024-08-30

**Authors:** Dong Hwan Ho, Hyejung Kim, Daleum Nam, Mi Kyoung Seo, Sung Woo Park, Dong-Kyu Kim, Ilhong Son

**Affiliations:** 1 InAm Neuroscience Research Center, Sanbon Medical Center, College of Medicine, Wonkwang University, 321, Sanbon-ro, Gunpo-si 15865, Republic of Korea; ceci776@naver.com (H.K.); ekfma304@naver.com (D.N.); 2 Paik Institute for Clinical Research, Inje University College of Medicine, Busan-si 47392, Republic of Korea; first1011486@hanmail.net (M.K.S.); neuro109@hanmail.net (S.W.P.); 3 Department of Convergence Biomedical Science, Inje University College of Medicine, Busan-si 47392, Republic of Korea; 4 Department of Ophthalmology, Yeouido St. Mary’s Hospital, College of Medicine, The Catholic University of Korea, 10, 63-Ro, Yeongdeungpo-Gu, Seoul 07345, Republic of Korea; rockbottom23@hanmail.net; 5 Sanbon Medical Center, Department of Neurology, College of Medicine, Wonkwang University, 321, Sanbon-ro, Gunpo-si 15865, Republic of Korea

**Keywords:** Parkinson’s disease, α-synuclein, leucine-rich repeat kinase 2, neuroinflammation, *Padina arborescens*

## Abstract

Leucine-rich repeat kinase 2 (LRRK2) and α-synuclein are involved in the pathogenesis of Parkinson’s disease. The activity of LRRK2 in microglial cells is associated with neuroinflammation, and LRRK2 inhibitors are crucial for alleviating this neuroinflammatory response. α-synuclein contributes to oxidative stress in the dopaminergic neuron and neuroinflammation through Toll-like receptors in microglia. In this study, we investigated the effect of the marine alga *Padina arborescens* on neuroinflammation by examining LRRK2 activation and the aggregation of α-synuclein. *P. arborescens* extract inhibits LRRK2 activity in vitro and decreases lipopolysaccharide (LPS)-induced LRRK2 upregulation in BV2, a mouse microglial cell line. Treatment with *P. arborescens* extract decreased tumor necrosis factor-α (TNF-α) gene expression by LPS through LRRK2 inhibition in BV2. It also attenuated TNF-α gene expression, inducible nitric oxide synthase, and the release of TNF-α and cellular nitric oxide in rat primary microglia. Furthermore, *P. arborescens* extract prevented rotenone (RTN)-induced oxidative stress in primary rat astrocytes and inhibited α-synuclein fibrilization in an in vitro assay using recombinant α-synuclein and in the differentiated human dopaminergic neuronal cell line SH-SY5Y (dSH). The extract increased lysosomal activity in dSH cells. In addition, *P. arborescens* extract slightly prolonged the lifespan of *Caenorhabditis elegans*, which was reduced by RTN treatment.

## 1. Introduction

Parkinson’s disease (PD) is the second-most common neurodegenerative disease [1]. Loss of motor activity is a primary manifestation of PD [2], and the degeneration of dopaminergic neurons is a key PD pathological feature [3]. Although microglia are necessary to maintain neuronal activity [4], neuroinflammation induced by reactive microglia is associated with PD progression [5]. Considering pro-inflammatory cytokine release from reactive microglia damages dopaminergic neurons, the mitigation of neuroinflammation can assist in PD therapy [6]. 

Leucine-rich repeat kinase 2 (LRRK2) plays a crucial role in the pathogenesis of PD [7]. Its activity is involved in several pathological mechanisms, including mitochondrial dysfunction [8], abnormal vesicle trafficking [9], aberrant autophagy–lysosome pathways [10], and neuroinflammation [11]. In a previous study, we demonstrated that inhibiting LRRK2 activity in reactive microglia can prevent dopaminergic neuronal degeneration [6]. In recent years, interest in LRRK2 inhibitors as crucial pharmacological agents for PD therapy has increased. 

α-Synuclein is a major component of the Lewy body, which is a key pathological feature of PD [12]. Studies have shown that the propagation of α-synuclein in the brain causes oxidative stress and neuroinflammation in the substantia nigra [13,14,15]. The aggregation of α-synuclein is promoted by the failure of lysosomal degradation [16]. Therefore, targeting the blockage and clearance of α-synuclein aggregates is considered a pivotal therapeutic target for PD [17,18]. 

In this study, we aimed to elucidate the therapeutic effects of marine algae on the symptoms associated with LRRK2 kinase activity and α-synuclein aggregation. A previous study reported that *Padina arborescens* exhibited protective effects against oxidative stress induced by high glucose levels in human umbilical vein endothelial cells (HUVECs) [19]. The increase in oxidative stress, such as reactive oxygen species (ROS) and nitric oxide (NO), caused by high glucose levels in HUVECs, is associated with neuroinflammation through reactive microglia [4]. Therefore, in this study, we investigated the effect of *P. arborescens* on synucleinopathy in the human dopaminergic cell line and neuroinflammation in the mouse microglial cell line BV2, as well as in rat primary microglia and astrocytes.

## 2. Materials and Methods

### 2.1. Cell Culture and Reagent Treatments

The mouse microglia secondary cell line BV2 was maintained in growth medium comprising Dulbecco’s modified Eagle’s medium (DMEM; LM001-07; Welgene, Gyeongsan-si, Republic of Korea) with 5% EqualFETAL (EF-0500-A; Atlas Biologicals, Inc., Fort Collins, CO, USA) and 1× antibiotic–antimycotic solution (15240062; Gibco, Carlsbad, CA, USA) in an incubator at 37 °C with 5% CO_2_. Lipopolysaccharide (LPS; 200 ng/mL; L4391, Sigma-Aldrich, St. Louis, MO, USA) mixed in EqualFETAL-depleted growth media was used to induce pro-inflammatory cytokines for 4 or 18 h. Simultaneously, the cells were co-treated with 1 μg/mL of *P. arborescens* extract (PA_EXT). 

The human dopaminergic neuronal cell line SH-SY5Y was maintained in DMEM (LM001-07; Welgene) with 10% EqualFETAL (EF-0500-A; Atlas Biologicals, Inc.) and 1× antibiotic–antimycotic solution (15240062; Gibco) in an incubator at 37 °C with 5% CO_2_. The differentiation of SH-SY5Y was induced via exposure to 10 μM all-trans retinoic acid (R2625, Sigma-Aldrich) for seven days, and treatment with the vehicle dimethyl sulfoxide (DMSO) and PA_EXT was maintained for 48 h.

### 2.2. Rat Primary Microglia and Astrocyte Preparation and Culture

For culturing, rat primary microglia and astrocytes were obtained from dissected fetal brains (embryonic day 17 [E17]) where the cortex was isolated. Tissues were incubated with trypsin–ethylenediaminetetraacetic acid (EDTA, 0.25%), phenol red (25200056; Gibco), and deoxyribonuclease I isolated from bovine pancreatic tissue (DN-25-100MG; Sigma-Aldrich) for 30 min and dissociated by pipetting. Cell debris and myelin were removed via centrifugation at 200× *g* for 10 min at room temperature (RT). The cells were subsequently passed through a nylon mesh filter and seeded into a T75 flask (11090; SPL, Pocheon-si, Republic of Korea), then maintained in 10% fetal bovine serum (FBS; BFS-1000; T&I, Chuncheon-si, Republic of Korea) and 1× antibiotic–antimycotic solution in DMEM/F-12 (LM002-04; Welgene). After one week, the cells were detached using TrypLE™ Express Enzyme (1×) with no phenol red (12604013; Gibco) and reseeded on a T75 flask to replicate. After two weeks, microglia were detached via rough tapping, while astrocytes were detached using TrypLE™ Express Enzyme (1×) with no phenol red. Next, 5 × 10^5^ cells of rat primary microglia or astrocytes were seeded in a 12-well plate (30012; SPL) for subsequent experiments. We administered 100 ng/mL of LPS or 0.8 μg/mL PA_EXT mixed in FBS-depleted growth media for 24 h, and then the cells and culture media were harvested for subsequent analyses.

### 2.3. In Vitro Kinase Assay

A mixture of 65 ng of full-length LRRK2 G2019S recombinant protein (A15200; Thermo Fisher Scientific, Waltham, MA, USA) and 50 μM adenosine triphosphate (ATP, A1852-1VL; Sigma-Aldrich) in 1× kinase buffer (PV3189; Thermo Fisher Scientific) was incubated at 35 °C for 1 h. Then, 4× Laemmli sample buffer (L1100-001; GenDEPOT, Katy, TX, USA) was added to stop the reaction, and the phosphorylation level of full-length LRRK2 G2019S was evaluated using Western blotting. 

### 2.4. Western Blot Analysis

To quantify proteins, the cells were washed twice with ice-cold Dulbecco’s phosphate-buffered saline (DPBS LB001-02; Welgene), harvested, and then mixed with 1× sample buffer diluted with 4× Laemmli sample buffer, reducing agent (GenDEPOT), and sterile water. The samples were sonicated for 20 s with a 10% amplification frequency using an Ultrasonic Processor (VCX 130; Sonics & Materials, Inc., Newtown, CT, USA) and boiled at 95 °C for 5 min. Each sample was loaded onto a 4–20% MINI-PROTEAN^®^ TGX Precast Protein Gel well (15 μL; 4561096; Bio-Rad, Hercules, CA, USA), and electrophoresis was performed at 100 V for 100 min. The proteins were transferred onto a nitrocellulose membrane (10600004; Cytiva, Marlborough, MA, USA) following sodium dodecyl sulfate–polyacrylamide gel electrophoresis at 300 mA for 80 min. Nitrocellulose membranes containing the proteins of interest were soaked in a mixture comprising 5% skim milk and Tris-buffered saline containing 0.1% Tween-20 (TBST) for 30 min at RT. The primary antibodies, listed in Table 1, were mixed overnight with 1% bovine serum albumin (BSA) in TBST.

The membranes were washed thrice with TBST and incubated with secondary antibodies (Table 1) to detect the protein bands. Luminata Crescendo Western horseradish-peroxidase (HRP, WBLUR0500; Merck & Co., Inc., Kenilworth, NJ, USA) was used to develop immunoreactive signals on the nitrocellulose membrane, and a MicroChemi 4.2 camera (Shimadzu, Kyoto, Japan) was used to obtain images of the protein bands. Full Western blot images can be found by File S1.

### 2.5. Messenger RNA (mRNA) Isolation and Complementary DNA (cDNA) Synthesis

The cells were washed twice with ice-cold DPBS, and the RNeasy Plus Mini kit (74134; Qiagen, Germantown, MD, USA) was used to isolate mRNA from harvested cells. For cDNA synthesis, we used the TORscript cDNA synthesis kit (EZ005S; Enzynomics, Daejeon, Republic of Korea). To synthesize and amplify cDNA, mixtures consisting of 2 µL 10× TOR script RT buffer, 1 µL TOR script Reverse Transcriptase (200 U/µL), 2 µL dNTP mixture (2 mM), 2 µL total RNA, 1 µL oligo (dT), 0.5 µL RNase inhibitor (40 U/µL), 11.5 µL RNase-free sterile water, and 1 µg of total RNA were incubated at 55 °C for 60 min. To stop the reaction, samples were incubated at 95 °C for 5 min.

### 2.6. Quantitative-Polymerase Chain Reaction (qPCR) Analysis

qPCR analyses were performed using 0.5 μL of synthesized cDNA, 5 μL of TOPreal™ SYBR Green qPCR Premix (RT500S; Enzynomics, Daejeon, Republic of Korea), 0.25 μL of primers, and 4.25 μL of RNase-free sterile water, with the primers listed in Table 2. 

The analysis was performed using a magnetic induction cycler (MIC; BioMolecular Systems, Upper Coomera, QLD, Australia). mRNA levels were determined using the following equation: 2^−ΔΔCT^. 

### 2.7. Enzyme-Linked Immunosorbent Assays (ELISAs) of Cytokines and Neurotropic Factors

We harvested the culture media of BV2 cells and rat primary microglia via centrifugation at 4000 rpm for 10 min at 4 °C to detect the levels of pro-inflammatory cytokines, such as tumor necrosis factor-alpha (TNF-α) and inducible nitric oxide (iNOS). One hundred microliters of culture media from each experiment was analyzed using the rat TNF-α ELISA kit (DY510-05; R&D System, Minneapolis, MN, USA). All assays were performed according to the manufacturer’s instructions. A Synergy 2 microplate reader (Biotek Instruments, Inc., Winooski, VT, USA) was used to measure the absorbance at 450 nm.

### 2.8. Measurement of Cellular NO Levels

The Griess assay (G7921; Thermo Fisher Scientific), which assesses the two primary stable and nonvolatile breakdown products of NO, was used to measure the NO levels. Fifty microliters of cell lysate supernatant was collected following centrifugation at 4000 rpm for 10 min at 4 °C and subjected to the Griess assay. Equal volumes of N-(1-naphthyl) ethylenedi-amine (Component A) and sulfanilic acid (Component B) were mixed to obtain the Greiss reagent, 20 μL of which was mixed with the deionized water-mixed sample (280 μL) in a dark 96-well plate. After 30 min of incubation at RT, the absorbance of the samples was recorded at 548 nm using the Synergy^TM^ 2 system (Biotek).

### 2.9. Detection of Cellular ROS 

Rat primary astrocytes seeded in a dark 96-well plate were treated with 3 μM rotenone (RTN; 557368; Sigma-Aldrich) or DMSO (D2650, Sigma-Aldrich) solutions and co-treated with 1 μg/mL PA_EXT. After 24 h, 5 μM CellRox (C10444; Thermo Fisher Scientific) and 2 μM Hoechst 33342 (62249; Thermo Fisher Scientific) solutions were added, and the cells were incubated for 30 min. The cells were then washed twice with ice-cold DPBS and fixed with ice-cold 4% paraformaldehyde (161-20141; Fujifilm Wako Pure Chemical Corporation, Tokyo, Japan) for 15 min. Next, the cells were then washed thrice with ice-cold DPBS, and the absorbances of CellRox and Hoechst 33342 were recorded at excitation wavelengths of 485 and 361 nm and emission wavelengths of 520 and 497 nm, respectively, using a FlexStation 3 multi-mode microplate reader (Molecular Devices, San Jose, CA, USA). 

### 2.10. Seed-Accelerated Fibrilization of α-Synuclein

Recombinant α-synuclein monomer was purified and isolated from BL21 *Escherichia coli* transformed with a human α-synuclein plasmid, which was provided by Dr. Seung-Jae Lee. Next, 1 mg/mL of monomeric α-synuclein was incubated with or without 5 μg/mL of fibrillar α-synuclein (fibril seed) at 37 °C with shaking at 150 rpm. We added the vehicle DMSO or PA_EXT to the pool of monomeric α-synuclein with fibril seeds during incubation at the indicated time.

### 2.11. Thioflavin T Assay

At the indicated time, each sample (100 μL) was collected and promptly frozen to complete the thioflavin T assay. We followed the procedures described previously [20]. 

### 2.12. Sandwich ELISA for α-Synuclein

Differentiated SH-SY5Y (dSH) cells treated with DMSO or PA_EXT were lysed with phosphate-buffered saline (PBS; Gibco, 18912014) containing 1% Triton-X 100 (85111, Thermo Fisher Scientific) and a 1× Xpert protease inhibitor cocktail (P3100-005, GenDEPOT). Lysates were centrifuged at 12,000× *g* at 4 °C for 10 min, and the supernatants were subjected to the sandwich ELISA, as described in a previous study [21]. 

### 2.13. Analysis of Lysosomal Activity

The differentiated SH-SY5Y (dSH) cells treated with DMSO or PA_EXT were stained with 1 μM LysoTracker Blue DND-22 (L7525; Thermo Fisher Scientific), 2 μM 5-(Pentafluorobenzoylamino) Fluorescein Di-β-D-Glucopyranoside (PFB-FDGlu, P11947; Thermo Fisher Scientific), and 0.5 μM SYTO 59 Red Fluorescent Nucleic Acid Stain (S11341; Thermo Fisher Scientific) for 1 h. After washing twice with PBS, images of cells were acquired using the FLoid™ Cell Imaging Station (4471136, Thermo Fisher Scientific). The intensity was analyzed using the Multi Gauge V3.0 software program (Fujifilm, Tokyo, Japan).

### 2.14. Cathepsin D Activity

Cathepsin D activity assay kit (ab63502, Abcam) was used for the analysis, and we followed the instruction of manufacturer. 

### 2.15. Culturing of Worms

All worms were cultivated and preserved on nematode growth medium (NGM) plates seeded with the *E. coli* strain OP50 at 20 °C using standard processes [22]. Wild-type Bristol N2 worms were acquired from the Caenorhabditis Genetics Center (CGC; University of Minnesota, St. Paul, MN, USA) for analysis. 

### 2.16. Lifespan Assay of C. elegans

Lifespan analyses were performed using the synchronization method, as previously described [23]. Age-synchronized L4-stage worms grown from the eggs produced by gravid worms were transferred to NGM plates containing 10 μM 5-fluoro-2′-deoxyuridine (FudR; Sigma-Aldrich, F0503). The numbers of live and dead worms were determined and recorded regularly. Worms that ruptured, burrowed, or crawled off the plates were included in the lifespan assay as censored animals. The survival rate was determined using GraphPad Prism version 10.2.0 (GraphPad Software Inc., San Diego, CA, USA), and the mean lifespan was assessed using OASIS 2 (Online Application for Survival Analysis 2; https://sbi.postech.ac.kr/oasis2/, accessed on 10 July 2024) [24].

### 2.17. Data Estimation and Statistical Analyses 

Western blot images were densitometrically analyzed using Multi Gauge image analysis software (Fujifilm, Tokyo, Japan), and the significance and construction of data charts were estimated using Prism 8 software (GraphPad, San Diego, CA, USA). The statistical analyses and significance levels used in this study are indicated in the figure legends. 

## 3. Results

### 3.1. PA_EXT Inhibits LRRK2 Activity in BV2 Cells In Vitro

**Figure 1:**
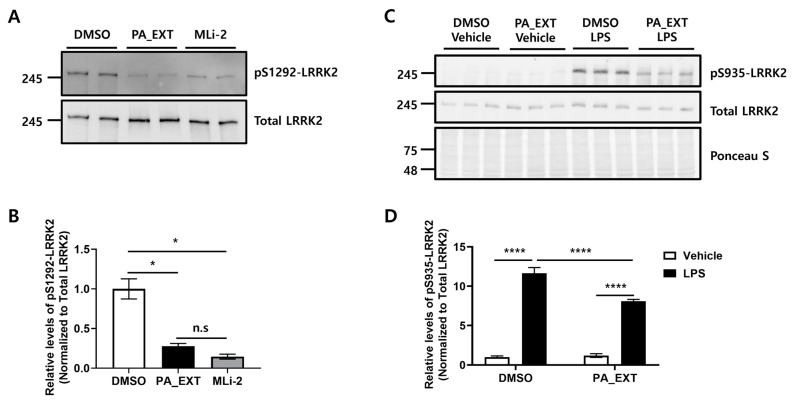
PA_EXT inhibits LRRK2 activity. (**A**) PA_EXT and a commercially available LRRK2 inhibitor, MLi-2, were tested for their effects on autophosphorylation at the serine 1292 site (pS1292) of the LRRK2 G2019S recombinant protein. Densitometry reading of pS1292 was normalized to total LRRK2 densitometry reading, and each test group was compared to the control (DMSO). (**B**) S1292 autophosphorylation levels were analyzed and graphed. One-way analysis of variance (ANOVA) with Bonferroni’s multiple comparison test was used, *n* = 2, *; *p* < 0.05. n.s; not significant. (**C**) LRRK2 activity changes after treatment with 200 ng/mL LPS or 1 μg/mL PA_EXT for 18 h in BV2 cells were assessed by the levels of phosphorylation at the serine 935 site (pS935) in mice using Western blot analysis. (**D**) S935 phosphorylation levels were analyzed and graphed. The densitometry reading of pS935 was normalized to the total LRRK2 densitometry reading, and each group was compared to the controls (DMSO and vehicle). A two-way ANOVA with Bonferroni’s multiple comparison test was used, n = 3, *; *p* < 0.05, ****; *p* < 0.0001.

To verify LRRK2 inhibition by PA_EXT, we performed an in vitro LRRK2 assay and assessed LPS-induced neuroinflammation in BV2 cells, which are appropriate surrogates for upregulating LRRK2 in the cell system. PA_EXT significantly inhibited LRRK2 activity, thereby decreasing autophosphorylation at the S1292 site of the LRRK2 G2019S recombinant protein (Figure 1A,B). The extent of LRRK2 inhibition by PA_EXT was comparable to that achieved with the LRRK2 inhibitor MLi-2 (Figure 1A,B). Additionally, PA_EXT significantly inhibited LRRK2 activity, consistent with the phosphorylation of the pS935 site of mouse LRRK2 by LPS in BV2 cells. However, PA_EXT blocked the LPS-induced increase in LRRK2 activity (Figure 1C,D). These results suggest that further research is needed to clarify the pharmacological activity of *P. arborescens* against LRRK2.

### 3.2. PA_EXT Decreases TNF-α Expression by Inhibiting LRRK2 Activity in BV2 Cells

**Figure 2:**
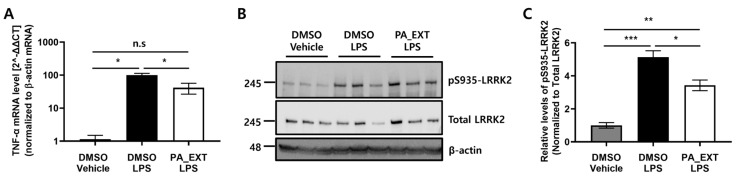
PA_EXT reduces pro-inflammatory cytokines in BV2 cells. (**A**) TNF-α gene expression levels after treatment with 200 ng/mL LPS or 1 μg/mL PA_EXT for 4 h were measured using qPCR and compared to the controls (DMSO and vehicle). (**B**) The phosphorylation of LRRK2 in BV2 was analyzed using Western blot analysis. (**C**) Graph of LRRK2 phosphorylation data. The densitometry reading of pS935 was normalized to that of total LRRK2 and compared to the controls (DMSO and vehicle). One-way ANOVA with Bonferroni’s multiple comparison test was used, *n* = 3, *; *p* < 0.05, **; *p* < 0.01, ***; *p* < 0.001, n.s; not significant.

To confirm the reduction in pro-inflammatory cytokines through LRRK2 inhibition by PA_EXT, we compared TNF-α mRNA levels between LPS treatment alone and co-treatment with LPS and PA_EXT. LPS treatment significantly elevated LRRK2 activity and increased TNF-α expression. Co-treatment with PA_EXT significantly reduced both LRRK2 activity and TNF-α expression (Figure 2A–C). These results indicate that the reduction in pro-inflammatory cytokine release is mediated by LRRK2 inhibition through a therapeutic component of *P. arborescens*.

### 3.3. PA_EXT Reduces the Gene Expression of TNF-α and iNOS in Rat Primary Microglia

**Figure 3:**
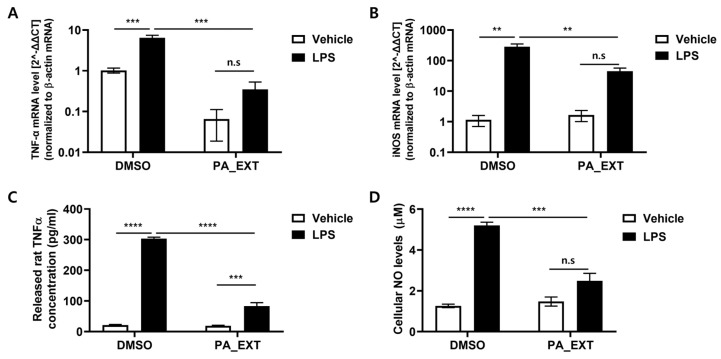
PA_EXT reduces pro-inflammatory cytokines in rat primary microglia. Rat primary microglia cells were treated with 100 ng/mL LPS or 0.8 μg/mL PA_EXT. Gene expression levels of TNF-α (**A**) and inducible iNOS (**B**) were analyzed using qPCR. (**C**) Supernatants of cell lysates were analyzed using a commercial rat TNF-α ELISA kit. (**D**) NO levels were measured using Greiss assay (**D**) for nitrite (NO−_2_) and nitrate (NO^−^_3_), the two stable products of NO. A two-way ANOVA with Bonferroni’s multiple comparison tests was used, *n* = 3, **; *p* < 0.01, ***; *p* < 0.001, ****; *p* < 0.0001, n.s; not significant.

To confirm that PA_EXT inhibits neuroinflammation in microglia, we examined its effect on LPS-induced neuroinflammation in primary rat microglia. We observed that PA_EXT significantly reduced TNF-α and iNOS expression induced by LPS treatment (Figure 3A,B). Additionally, the high levels of TNF-α (Figure 3C) and cellular NO (Figure 3D) released following the LPS treatment were decreased by PA_EXT co-treatment. These results indicate that *P. arborescens* may reduce neuroinflammation in microglia.

### 3.4. PA_EXT Alleviates Oxidative Stress in Rat Primary Astrocytes

**Figure 4:**
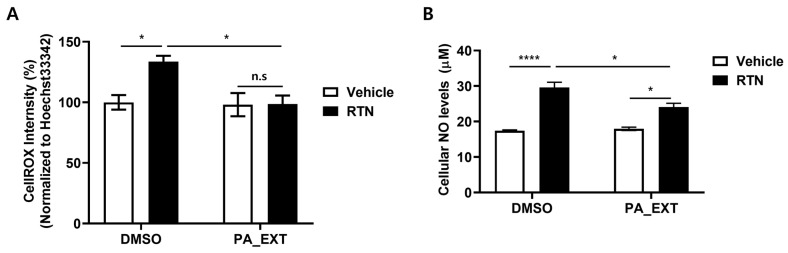
PA_EXT administration diminishes the oxidative stress caused by RTN treatment in rat primary astrocytes. Rat primary astrocytes were treated with 3 μM RTN or 1 μg/mL PA_EXT. (**A**) Cellular ROS (CellROX) levels were measured using fluorescence spectrophotometry. CellROX intensity was normalized to Hoechst33342 intensity and compared to controls (DMSO alone). (**B**) NO^−^₂ and NO^−^_3_ levels were measured using the Griess assay and graphed. A two-way ANOVA with Bonferroni’s multiple comparison test was used, *n* = 3, *; *p* < 0.05, ****; *p* < 0.0001, n.s; not significant.

Astrocytes are associated with neuroinflammation, and reactive astrocytes exhibit increased ROS and NO levels under neuroinflammatory conditions [25]. Therefore, we evaluated the effects of PA_EXT on the ROS and NO levels in rat primary astrocytes after RTN treatment. Cellular ROS and NO levels increased after RTN treatment; however, co-treatment with PA_EXT and RTN decreased these levels (Figure 4A,B). These results indicate that *P. arborescens* can be used both as an LRRK2 inhibitor and a neuroinflammatory regulator.

### 3.5. PA_EXT Blocks α-Synuclein Aggregation and Promotes Its Clearance in Differentiated SH-SY5Y (dSH) Cells

**Figure 5:**
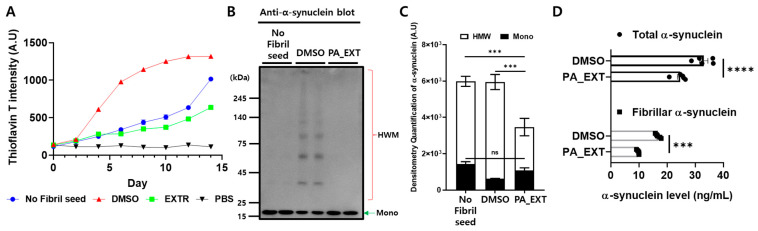
Blockage of α-synuclein aggregation by PA_EXT. α-synuclein fibrilization was examined using 1 μg/mL recombinant monomeric α-synuclein with 50 ng/mL α-synuclein fibril seeds or without α-synuclein fibril (no fibril seed), with DMSO as a vehicle control or 1 μg/mL PA_EXT. (**A**) The thioflavin T assay was used to quantify the β-sheet structures, which are abundant in fibrillar α-synuclein. PBS was tested as a control in the Thioflavin T assay. (**B**) Western blotting was used to assess α-synuclein aggregation. (**C**) Densitometry was used to quantify the high molecular weight of α-synuclein (HWM) and monomeric α-synuclein (Mono). A one-way ANOVA and Bonferroni’s multiple comparison tests were used, n = 1, duplication assay, ***; *p* < 0.001, ****; *p* < 0.0001, ns; not significant. SH-SY5Y cells were differentiated for 7 d, and DMSO or PA_EXT were administered for 7 d. (**D**) The supernatants of cell lysate were subjected to sandwich ELISA for estimation of total or fibrillar α-synuclein levels. A two-way ANOVA and Sidak’s multiple comparison tests were used, *n* = 5. ***; *p* < 0.001, ****; *p* < 0.0001.

**Figure 6:**
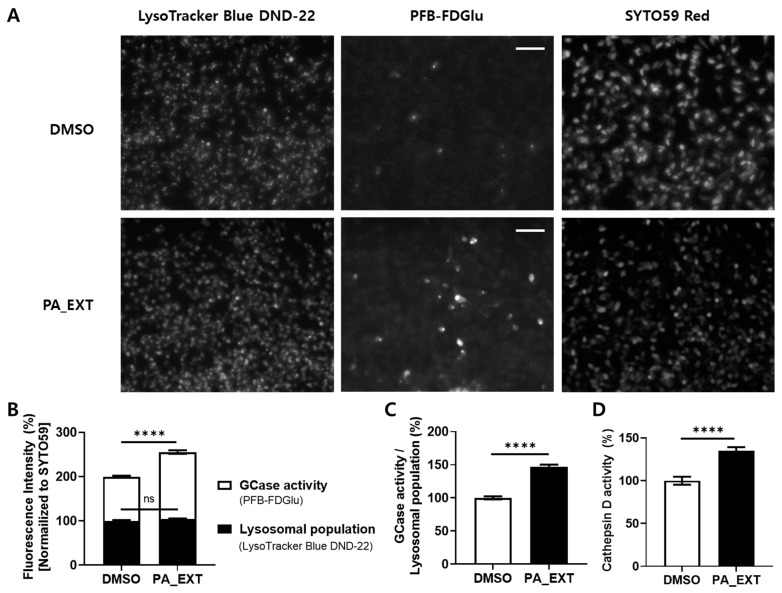
Elevation of lysosomal activity by PA_EXT in dSH cells. (**A**) Lysosomes in dSH cells were stained with LysoTracker Blue DND-22, and GCase activity was represented by the intensity of PFB-FDGlu, which is a substrate of glucocerebrosidase. All cells were also stained with SYTO 59 Red Fluorescent Nucleic Acid Stain (SYTO 59 Red). Cell images were captured at 460× optical zoom, and the right upper white bar on the PFB-FDGlu images (middle panels) indicates the scale bar (40 µm). (**B**) Lysosomal populations were measured via Lyso Tracker Blue DND-22 intensity, and GCase activity was estimated using PFB-FDGlu intensity. Fluorescence intensities were normalized to SYTO59 Red intensity. (**C**) The ratio of GCase activity to lysosomal population. (**D**) Cathepsin D activity was estimated in cell lysates. Student’s *t*-test was used, *n* = 4, ****; *p* < 0.0001, ns.; not significant.

α-synuclein aggregation is associated with oxidative stress. Since PA-EXT alleviates oxidative stress in rat primary astrocytes, we hypothesized that it might affect the aggregation of α-synuclein in dopaminergic neurons. We analyzed the seed-induced fibrilization of recombinant monomeric α-synuclein using α-synuclein fibril (fibril seed) with or without PA_EXT for 14 days. Samples collected every 2 days were analyzed using the thioflavin T assay at the end of incubation. The presence of seeds (DMSO) accelerated the fibrilization of α-synuclein fibrilization compared to the absence of seeds (no fibril seed). However, the addition of PA_EXT significantly prevented α-synuclein fibrilization (Figure 5A). No increase in thioflavin T was observed in the PBS control condition. To confirm the thioflavin T assay results, we used Western blotting on the remaining samples. DMSO-treated cells exhibited an increase in high-molecular-weight (HMW) α-synuclein and a decrease in monomeric α-synuclein compared to the no fibril seed condition. PA-EXT significantly blocked the generation of HMW α-synuclein compared to that in no fibril seed and DMSO treatments (Figure 5B,C). We also tested endogenous α-synuclein aggregation in a human dopaminergic neuronal cell line, SH-SY5Y, following retinoic acid (RA) differentiation to verify PA_EXT’s effect. PA_EXT inhibited the generation of fibrillar α-synuclein in dopaminergic neuronal cells. However, the levels of total α-synuclein in dopaminergic neuronal cells significantly decreased after PA_EXT exposure (Figure 5D). We hypothesized that this decrease was due to lysosomal degradation. As a previous study revealed that GCase, a selective lysosomal glucocerebrosidase, in lysosomes, is associated with the degradation of α-synuclein, we analyzed lysosomal GCase activity using PFB-FDGlu [26]. Although the lysosomal population following PA_EXT treatment was similar to that following DMSO treatment (Figure 6A,B), the GCase activity increased with PA_EXT (Figure 6A–C). Although the cytosolic GCase was detected by flow cytometry or spectrophotometry, GCase fluorescence primarily showed a punctate structure in the cell body rather than a diffused cytosolic pattern. To validate α-synuclein degradation by PA_EXT treatment via lysosomal activity, we tested cathepsin D enzymatic activity, which is involved in α-synuclein degradation. PA_EXT increased cathepsin D activity (Figure 6D). These results suggest that PA_EXT may be useful for blocking α-synuclein aggregation and enhancing the lysosomal degradation of α-synuclein in dopaminergic neurons.

### 3.6. PA_EXT Prolongs the Lifespan of C. elegans Damaged by RTN

**Figure 7:**
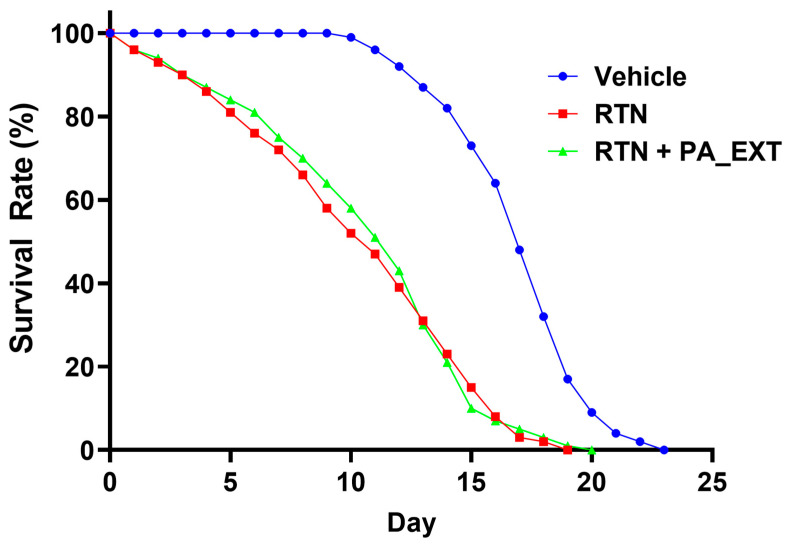
*C. elegans* lifespan analyses post-PA_EXT treatment. *C. elegans* co-supplemented with PA_EXT and RTN showed a marginal increase in mean lifespan compared to RTN-treatment alone. Life span analysis for each treatment was conducted once, with 100 worms used in each treatment group.

To clarify the therapeutic effect of PA_EXT in PD models, we analyzed the lifespan of *C. elegans* treated with RTN or co-treated with RTN and PA_EXT. Co-treatment with RTN and PA_EXT increased the lifespan by approximately half a day compared to the RTN treatment alone (Figure 7). At early ages (until day 12), PA_EXT reversed the decreased survival rate observed with the RTN treatment, though this effect did not persist. The protective effect of PA_EXT was not sufficient to fully rescue RTN-induced changes (Table 3). These results suggest that PA_EXT may be a possible substance for alleviating the toxicity caused by oxidative stress that occurs over a short period or temporarily. Further studies should validate these findings using other independent strains of *C. elegans*.

## 4. Discussion

LRRK2 activity is associated with PD pathogenesis [7]. Oxidative stress in the dopaminergic neurons is a pathological feature of PD [27]. The sources of oxidative stress vary and include mitochondrial dysfunction, autophagic accumulation, and lysosomal failure [28,29,30]. These cellular mechanisms are linked to the LRRK2 activity in dopaminergic neurons [8,10]. Additionally, LRRK2 is involved in neuroinflammation in microglia and astrocytes [11,31]. Our previous study showed that treatment with an LRRK2 inhibitor upregulated LRRK2 activity, which was upregulated by LPS in BV2 cells, rat primary microglia, and human microglial cell lines [8]. LRRK2 inhibitors attenuate the release of pro-inflammatory cytokines, thereby mitigating the damage to dopaminergic neurons [6]. As the LRRK2 inhibitor led to cellular stress, we attempted to identify a novel LRRK2 inhibitor that is devoid of cellular stress effects from a natural source. We analyzed herbs, plant seeds, and marine algal extracts and determined that *P. arborescens* exhibited the desired LRRK2 inhibitory features (Figure 1 and Figure 2A,B). Additionally, *P. arborescens* reduced the expression and release of pro-inflammatory cytokines in microglial cells (Figure 2C and Figure 3). ROS and NO production were decreased by PA_EXT treatment (Figure 3D and Figure 4). A previous study demonstrated that *P. arborescens* diminished high glucose-mediated oxidative stress in HUVECs [19]. Another study revealed that *P. arborescens* acts as an antagonist of membrane progestin receptor α (mPRα) [32], which is involved in protecting human dopaminergic neuronal cell lines against oxidative stress and aiding the regeneration of Schwann cell-like differentiated adipose stem cells [33]. These features agree with the findings of this study and suggest that *P. arborescens* is a promising therapeutic agent for PD. 

Since the loss of dopaminergic neurons in the substantia nigra pars compacta is a primary pathology of PD [3], we investigated the protective effects of *P. arborescens* on dopaminergic neurons. PA_EXT blocked the aggregation of α-synuclein in vitro and in dSH cells (Figure 5). Additionally, the lysosomal activity was elevated by PA_EXT, thereby preventing the accumulation of α-synuclein (Figure 6). Malfunctions in the autophagy–lysosomal pathway, vesicle trafficking, and mitochondrial membrane potential in dopaminergic neurons can aggravate neuronal vulnerability through oxidative stress, cellular senescence, and toxic environments in non-neuronal cells [3,9,10,27,28,29,30,34]. Therefore, further studies using PA_EXT should be performed with human dopaminergic neurons derived from reprogrammed stem cells under conditions of oxidative stress or cellular senescence. PA_EXT also slightly prolonged the lifespan of *C. elegans* exposed to RTN (Figure 7). However, additional strains of *C. elegans* should be tested, and further animal studies are needed to verify the protective role of *P. arborescens* in the whole brain tissue, including both neurons and non-neuronal cells.

The crude *P. arborescens* extract may contain numerous chemicals, peptides, proteins, DNA, RNA, and long non-coding RNA. Therefore, specific molecules must be identified, isolated, and carefully assessed for the desired purposes. A few of these molecules may affect LRRK2 activity, others may induce neurotrophic factors, and some may block the aggregation of α-synuclein, another major cause of PD progression [34]. 

In this study, we demonstrated that *P. arborescens* extract inhibits LRRK2 activity and mediates a decrease in neuroinflammatory responses in microglia. This study serves as a foundation for developing marine sources for pharmacological research on PD. However, further studies are needed to explore the effects of *P. arborescens* on dopaminergic neurons and to isolate its effective components. 

## 5. Conclusions

We found that *P. arborescens* presents promising avenues for the treatment of PD. It shows potential as an LRRK2 inhibitor, reduces neuroinflammation, and blocks α-synuclein aggregates. The results encourage further investigations into the therapeutic effects of *P. arborescens* on human dopaminergic neurons and astroglia. Additionally, there is a need to explore the overall protective role of *P. arborescens* in PD pathology. Future research should verify and expand the findings of this study using various animal models.

## 6. Patents

The research currently has a national patent application pending in the Republic of Korea (10-2024-0049552), and we have requested a foreign patent application process under the Patent Cooperation Treaty.

## Figures and Tables

**Table 1:** Antibodies used for Western blot analysis.

Antibody	Manufacturer and Catalog Number
Anti-pS1292 LRRK2	ab203181; Abcam, Cambridge, UK
Anti-pS935 LRRK2	ab133450; Abcam
Anti-LRRK2 (N241A/34)	75-253; NeuroMab, Davis, CA, USA
Anti-β-actin	sc-47778; Santa Cruz, Dallas, TX, USA
Anti-α-synuclein (Clone 42)	610787; BD Bioscience, San Jose, CA, USA
Peroxidase-conjugated AffiniPure goat anti-mouse IgG (H + L)	115-035-003; Jackson Immunoresearch Laboratories, Inc., West Grove, PA, USA
Peroxidase-conjugated AffiniPure goat anti-rabbit IgG (H + L)	115-035-144; Jackson Immunoresearch Laboratories, Inc.

**Table 2:** Primer sequences used in this study.

Genes			Sequence (5′-3′)
TNF-α	Rat	Forward	ACTGAACTTCGGGGTGATTG
		Reverse	GCTTGGTGGTTTGCTACGAC
iNOS (NOS2)		Forward	CACCTTGGAGTTCACCCAGT
		Reverse	ACCACTCGTACTTGGGATGC
β-actin		Forward	CAGGGTGTGATGGTGGGTATGG
		Reverse	AGTTGGTGACAATGCCGTGTTC
TNF-α	Mouse	Forward	CCGATGGGTTGTACCTTGTC
		Reverse	GCTGGGTAGAGAATGGATGAACA
β-actin		Forward	TGTTACCAACTGGGACGACA
		Reverse	TCTCAGCTGTGGTGGTGAAG

**Table 3:** Mean lifespan of *C. elegans*.

Group	Mean	S.E.M	N
Vehicle	16.05	0.28	100
RTN	9.38	0.47	100
RTN + PA_EXT	9.7	0.4433	100

## Data Availability

The datasets generated and/or analyzed in the current study are available from the corresponding author upon reasonable request.

## Supplementary Materials

The following are available online at https://www.mdpi.com/article/10.3390/neurosci5030024/s1, File S1: Full Western blot images.
