# Supplementary material for: Therapeutic Effect of Padina arborescens Extract on a Cell System Model for Parkinson’s Disease
Source: NeuroSci. 2024 Aug 30;5(3):301–14. doi: 10.3390/neurosci5030024 (PMC11469749; doi:10.3390/neurosci5030024)
Supplement: Supplementary file 1 [file neurosci-05-00024-s001.zip › neurosci-3127424-supplementary.pdf]

Figure 1A pS1292-LRRK2 blot

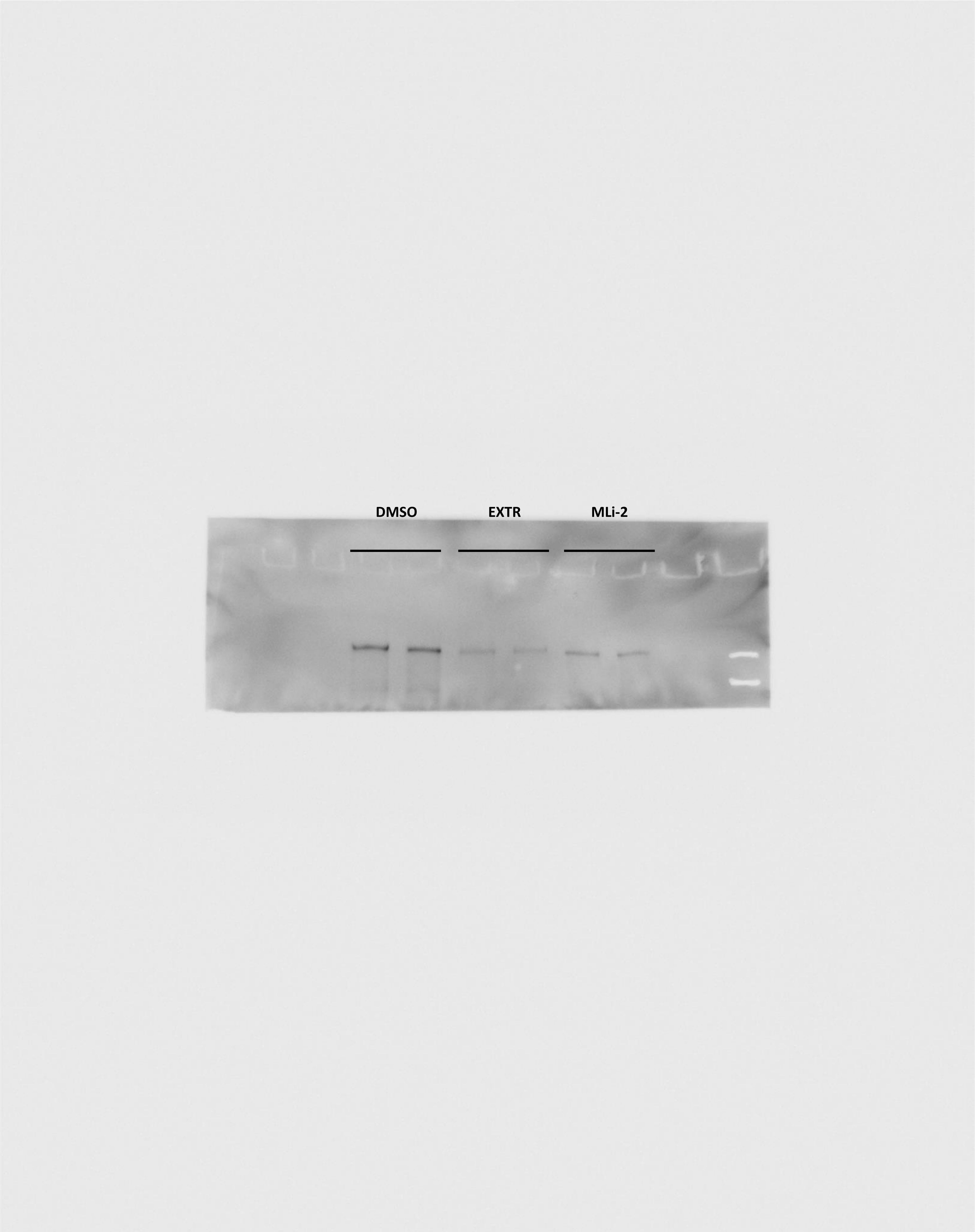

Figure 1A Total LRRK2 blot

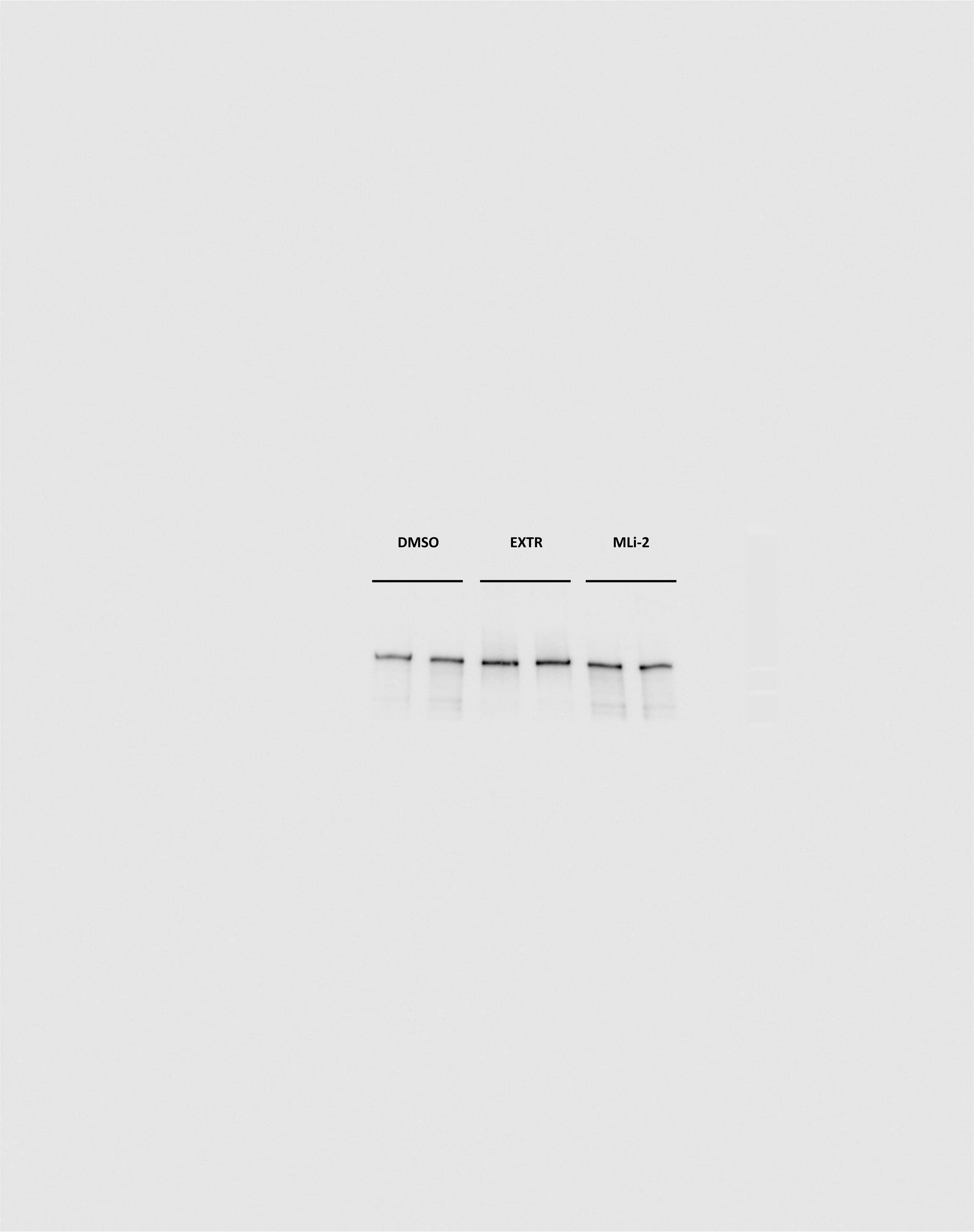

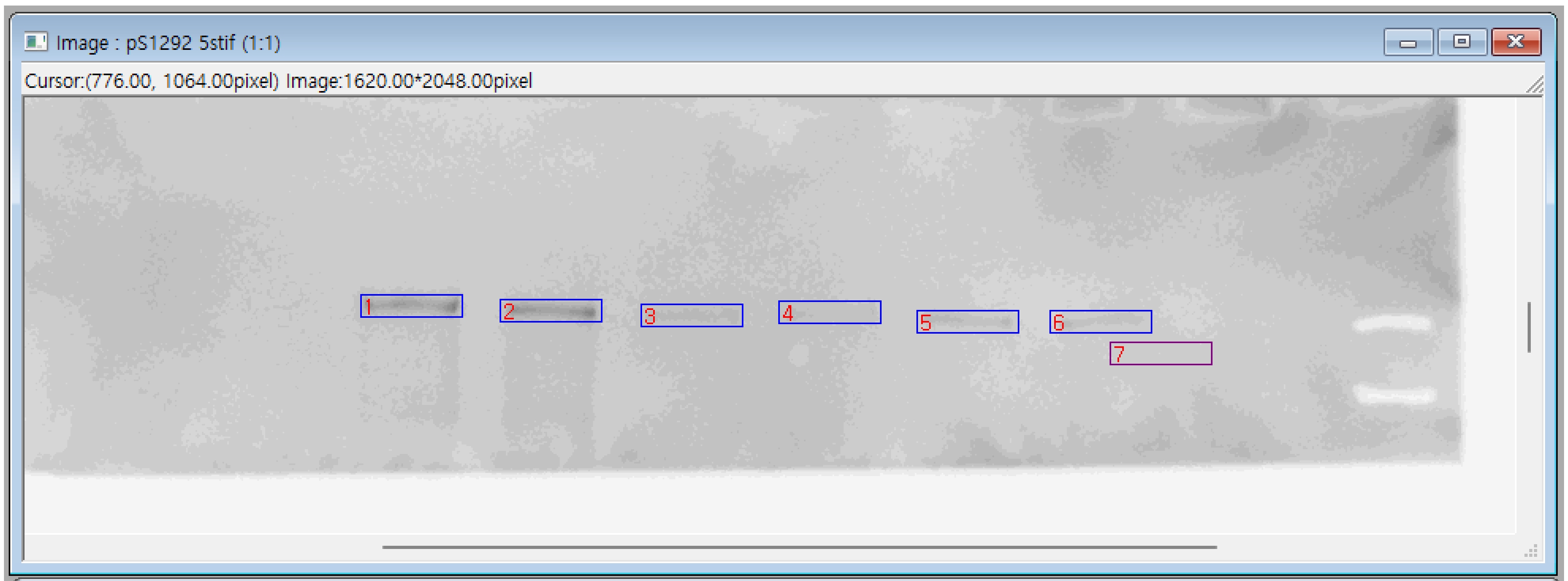

Result Table: pS1292 5stif [Quant] [Ratio(%): QL | Calibrated: ---]

Channel	No	Group	Index	Name	QL	Area(pixel2)	QL-BG	B	BG	Std	Ratio(%)	QL/pixel2	(Q-B)/pixel2	Calibrated
1	1	~	1		2030123.00	975.00	675094.00		1355029.00		19.54	2082.18	692.40	-------
1	2	~	2		2046270.00	975.00	691241.00		1355029.00		19.69	2098.74	708.97	-------
1	3	~	3		1721885.00	975.00	366856.00		1355029.00		16.57	1766.04	376.26	-------
1	4	~	4		1629520.00	975.00	274491.00		1355029.00		15.68	1671.30	281.53	-------
1	5	~	5		1520045.00	975.00	165016.00		1355029.00		14.63	1559.02	169.25	-------
1	6	~	6		1443204.00	975.00	88175.00		1355029.00		13.89	1480.21	90.44	-------
1	7	~	7		1355029.00	975.00	0.00	✓ B	1355029.00		-------	1389.77	0.00	-------

All Comparisons

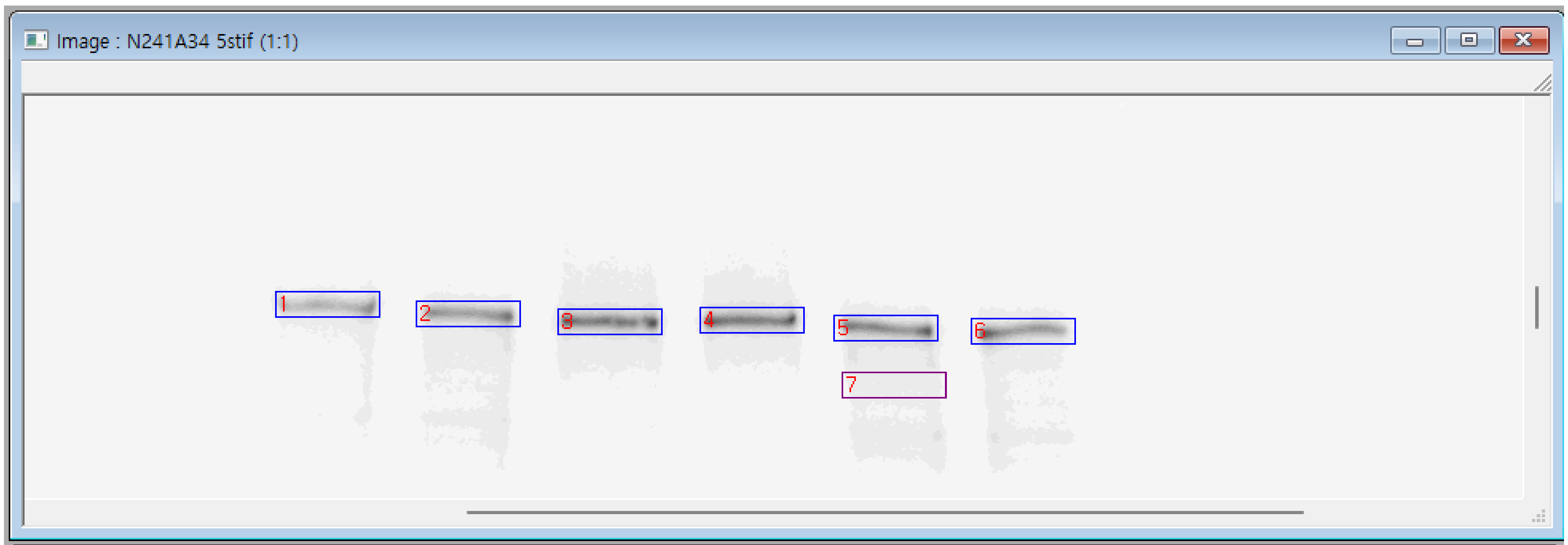

Result Table: N241A34 5stif [Quant] [Ratio(%): QL | Calibrated: ---]

Channel	No	Group	Index	Name	QL	Area(pixel2)	QL-BG	B	BG	Std	Ratio(%)	QL/pixel2	(Q-B)/pixel2	Calibrated
1	1	~	1		1420531.00	1122.00	778563.00		641968.00		13.08	1266.07	693.91	-------
1	2	~	2		1670853.00	1122.00	1028885.00		641968.00		15.38	1489.17	917.01	-------
1	3	~	3		2167851.00	1122.00	1525883.00		641968.00		19.95	1932.13	1359.97	-------
1	4	~	4		2109462.00	1122.00	1467494.00		641968.00		19.42	1880.09	1307.93	-------
1	5	~	5		1854691.00	1122.00	1212723.00		641968.00		17.07	1653.02	1080.86	-------
1	6	~	6		1640511.00	1122.00	998543.00		641968.00		15.10	1462.13	889.97	-------
1	7	~	7		641968.00	1122.00	0.00	✓ B	641968.00		-------	572.16	0.00	-------

All Comparisons

		pS1292	N241A	pS1292/N241A	
DMSO	1	692.4	693.91	0.997823925	1.126876
	2	708.97	917.01	0.773132245	0.873124
PA_EXT	1	376.26	1359.97	0.276667868	0.31245
	2	281.53	1307.93	0.215248522	0.243087
Mli-2	1	169.25	1080.86	0.156588272	0.17684
	2	90.44	889.97	0.101621403	0.114764

Figure 1C pS935-LRRK2 blot

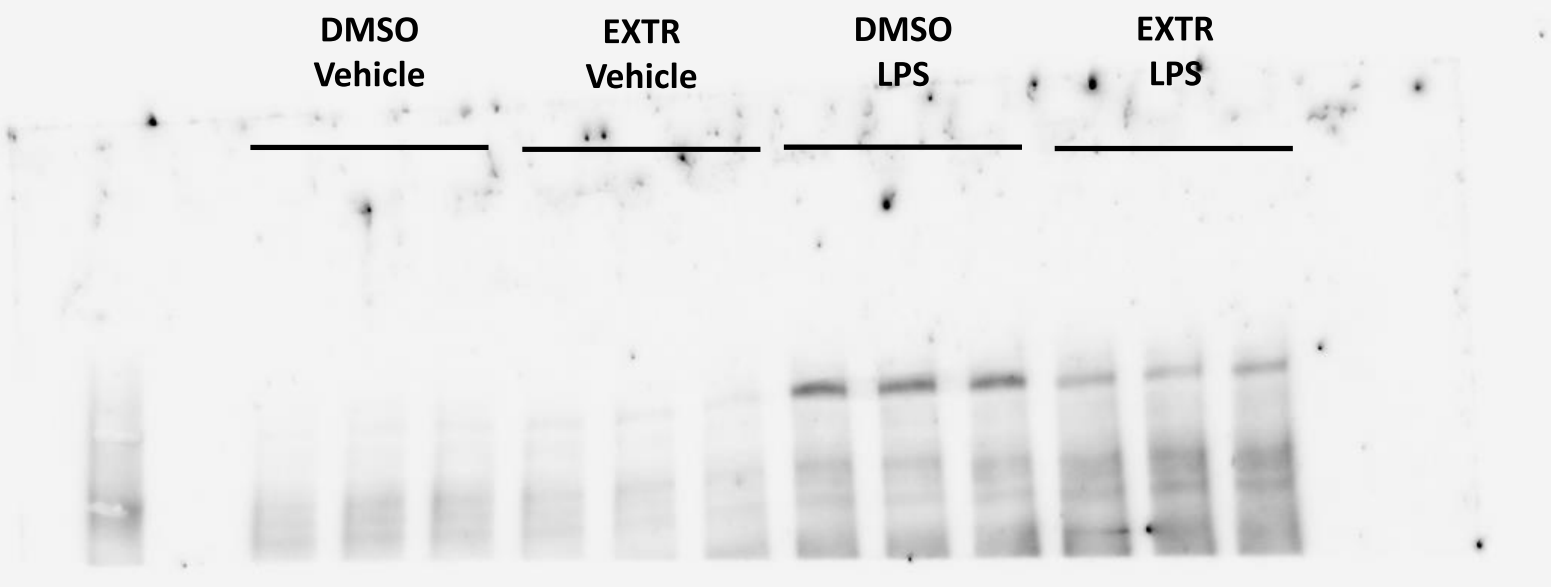

Figure 1C Total-LRRK2 blot

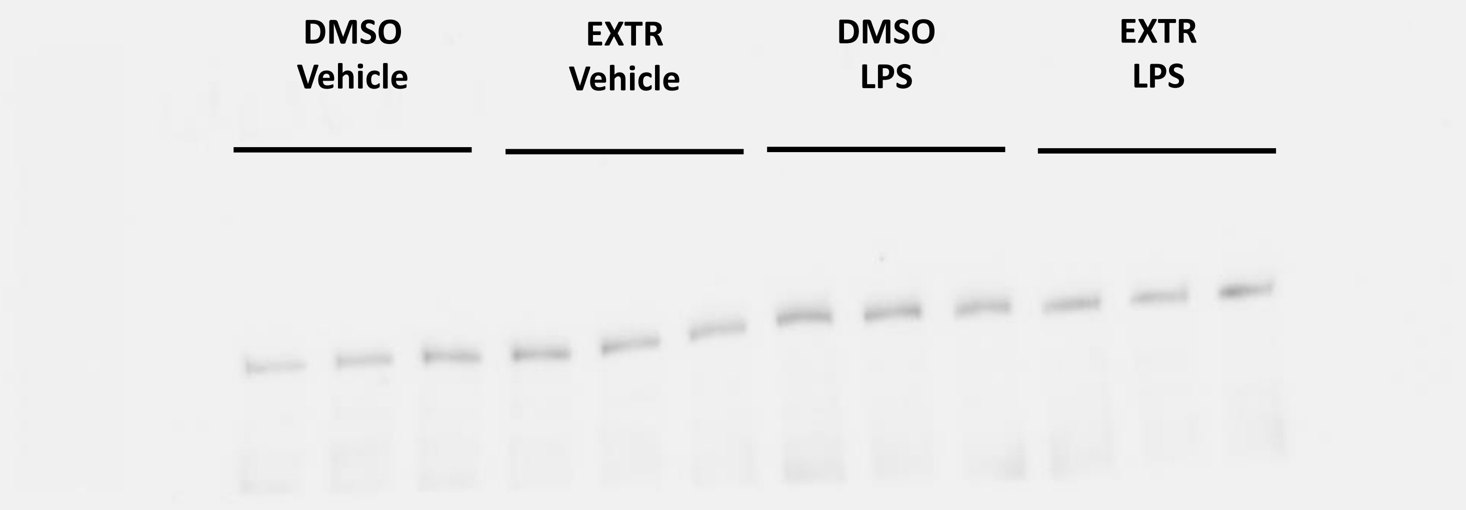

Figure 1C Ponceau S staining

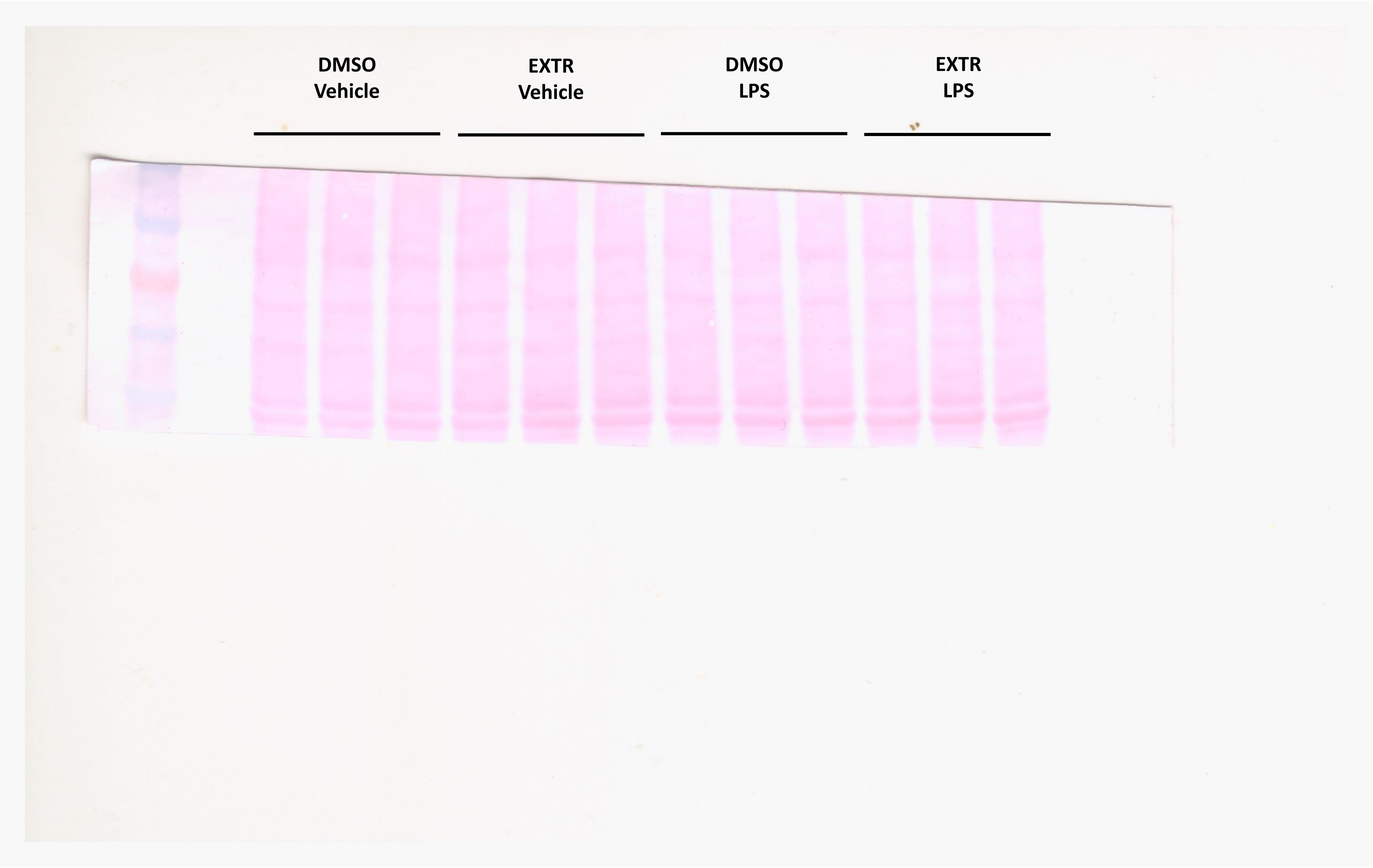

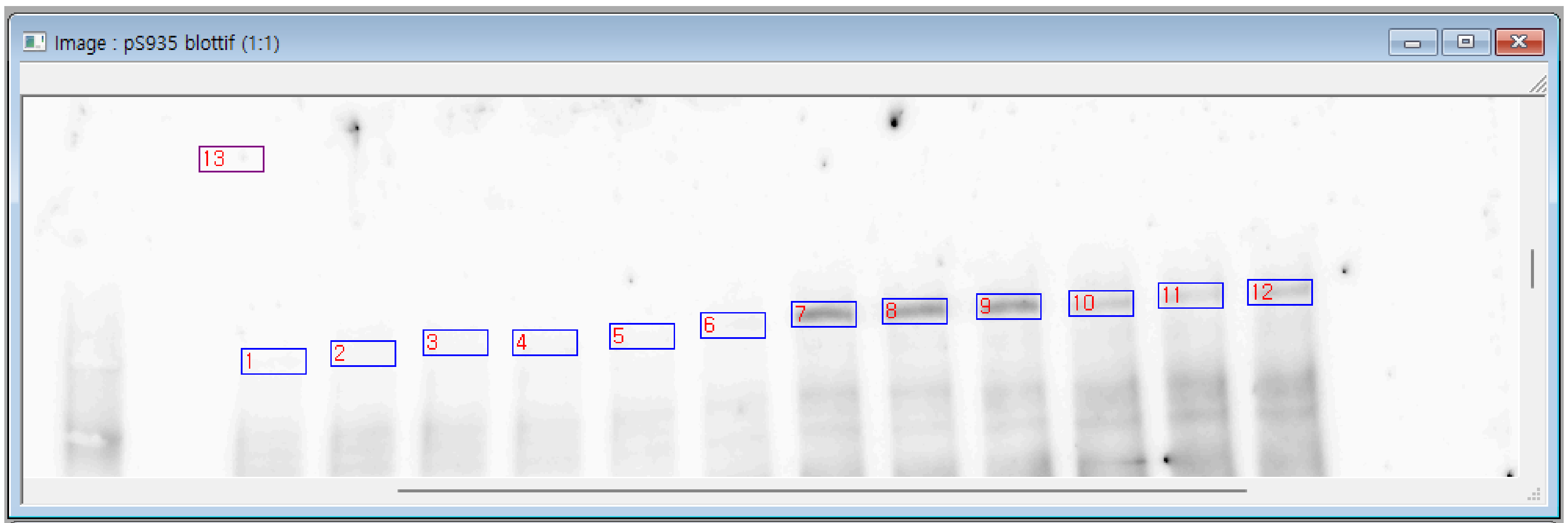

Result Table: pS935 blottif [Quant] [Ratio(%): QL | Calibrated: ---]

Channel	No	Group	Index	Name	QL	Area(pixel2)	QL-BG	B	BG	Std	Ratio(%)	QL/pixel2	(Q-B)/pixel2	Calibrated
1	1	~	1		367210.00	714.00	50083.00		317127.00		3.11	514.30	70.14	-------
1	2	~	2		419302.00	714.00	102175.00		317127.00		3.55	587.26	143.10	-------
1	3	~	3		427991.00	714.00	110864.00		317127.00		3.63	599.43	155.27	-------
1	4	~	4		431984.00	714.00	114857.00		317127.00		3.66	605.02	160.86	-------
1	5	~	5		430461.00	714.00	113334.00		317127.00		3.65	602.89	158.73	-------
1	6	~	6		480128.00	714.00	163001.00		317127.00		4.07	672.45	228.29	-------
1	7	~	7		1888437.00	714.00	1571310.00		317127.00		16.01	2644.87	2200.71	-------
1	8	~	8		1899547.00	714.00	1582420.00		317127.00		16.10	2660.43	2216.27	-------
1	9	~	9		1848932.00	714.00	1531805.00		317127.00		15.67	2589.54	2145.39	-------
1	10	~	10		1251602.00	714.00	934475.00		317127.00		10.61	1752.94	1308.79	-------
1	11	~	11		1068843.00	714.00	751716.00		317127.00		9.06	1496.98	1052.82	-------
1	12	~	12		1282289.00	714.00	965162.00		317127.00		10.87	1795.92	1351.77	-------
1	13	~	13		317127.00	714.00	0.00	✔ B	317127.00		-------	444.16	0.00	-------

All / Comparisons

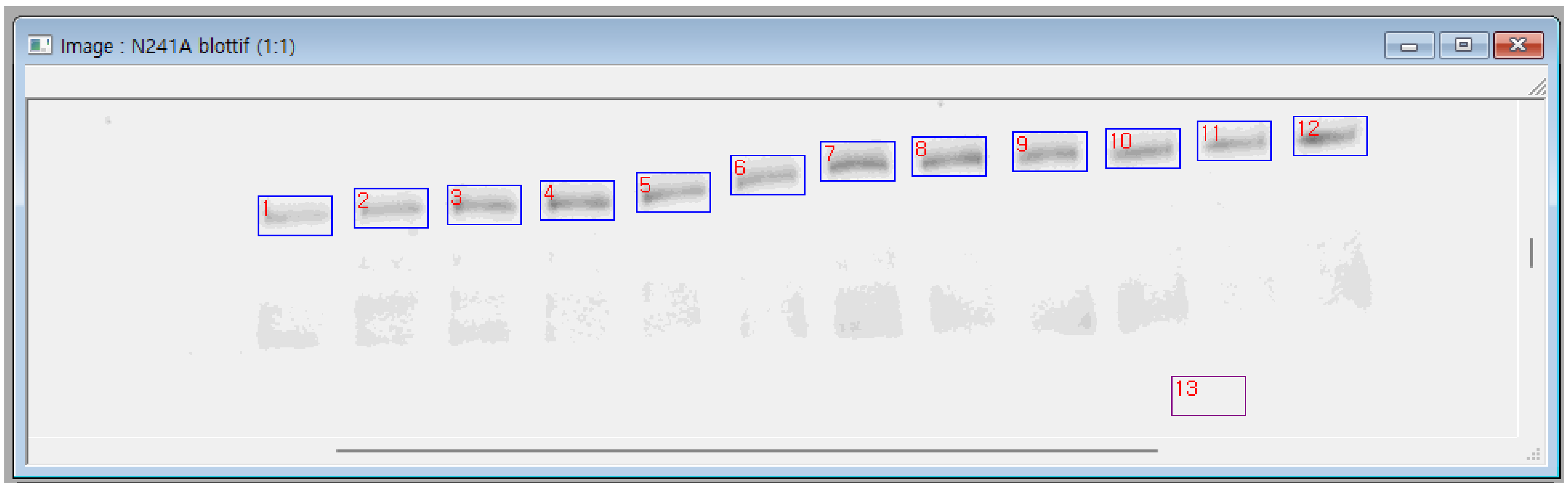

Result Table: N241A blottif [Quant] [Ratio(%): QL | Calibrated: ---]

Channel	No	Group	Index	Name	QL	Area(pixel2)	QL-BG	B	BG	Std	Ratio(%)	QL/pixel2	(Q-B)/pixel2	Calibrated
1	1	~	1		764162.00	1222.00	266149.00		498013.00		6.67	625.34	217.80	-------
1	2	~	2		832163.00	1222.00	334150.00		498013.00		7.27	680.98	273.45	-------
1	3	~	3		970060.00	1222.00	472047.00		498013.00		8.47	793.83	386.29	-------
1	4	~	4		1032604.00	1222.00	534591.00		498013.00		9.02	845.01	437.47	-------
1	5	~	5		949838.00	1222.00	451825.00		498013.00		8.29	777.28	369.74	-------
1	6	~	6		902416.00	1222.00	404403.00		498013.00		7.88	738.47	330.94	-------
1	7	~	7		1103903.00	1222.00	605890.00		498013.00		9.64	903.36	495.82	-------
1	8	~	8		1075436.00	1222.00	577423.00		498013.00		9.39	880.06	472.52	-------
1	9	~	9		982107.00	1222.00	484094.00		498013.00		8.57	803.69	396.15	-------
1	10	~	10		949661.00	1222.00	451648.00		498013.00		8.29	777.14	369.60	-------
1	11	~	11		884056.00	1222.00	386043.00		498013.00		7.72	723.45	315.91	-------
1	12	~	12		1007596.00	1222.00	509583.00		498013.00		8.80	824.55	417.01	-------
1	13	~	13		498013.00	1222.00	0.00	✔ B	498013.00		-------	407.54	0.00	-------

All / Comparisons

	pS935	N241A	pS935/N241A	
DMSO	70.14	217.8	0.322038567	0.774563
	143.1	273.45	0.52331322	1.258667
	155.27	386.29	0.401951901	0.96677
PA_EXT	160.86	437.47	0.367705214	0.8844
	158.73	369.74	0.429301671	1.032551
	228.29	330.94	0.689822929	1.659154
LPS	2200.71	495.82	4.438526078	10.67549
	2216.27	472.52	4.690319986	11.2811
	2145.39	396.15	5.415600151	13.02554
LPS+PA_EXT	1308.79	369.6	3.541098485	8.517008
	1052.82	315.91	3.332658036	8.015669
	1351.77	417.01	3.241576941	7.796602

Figure 2A pS935-LRRK2 blot

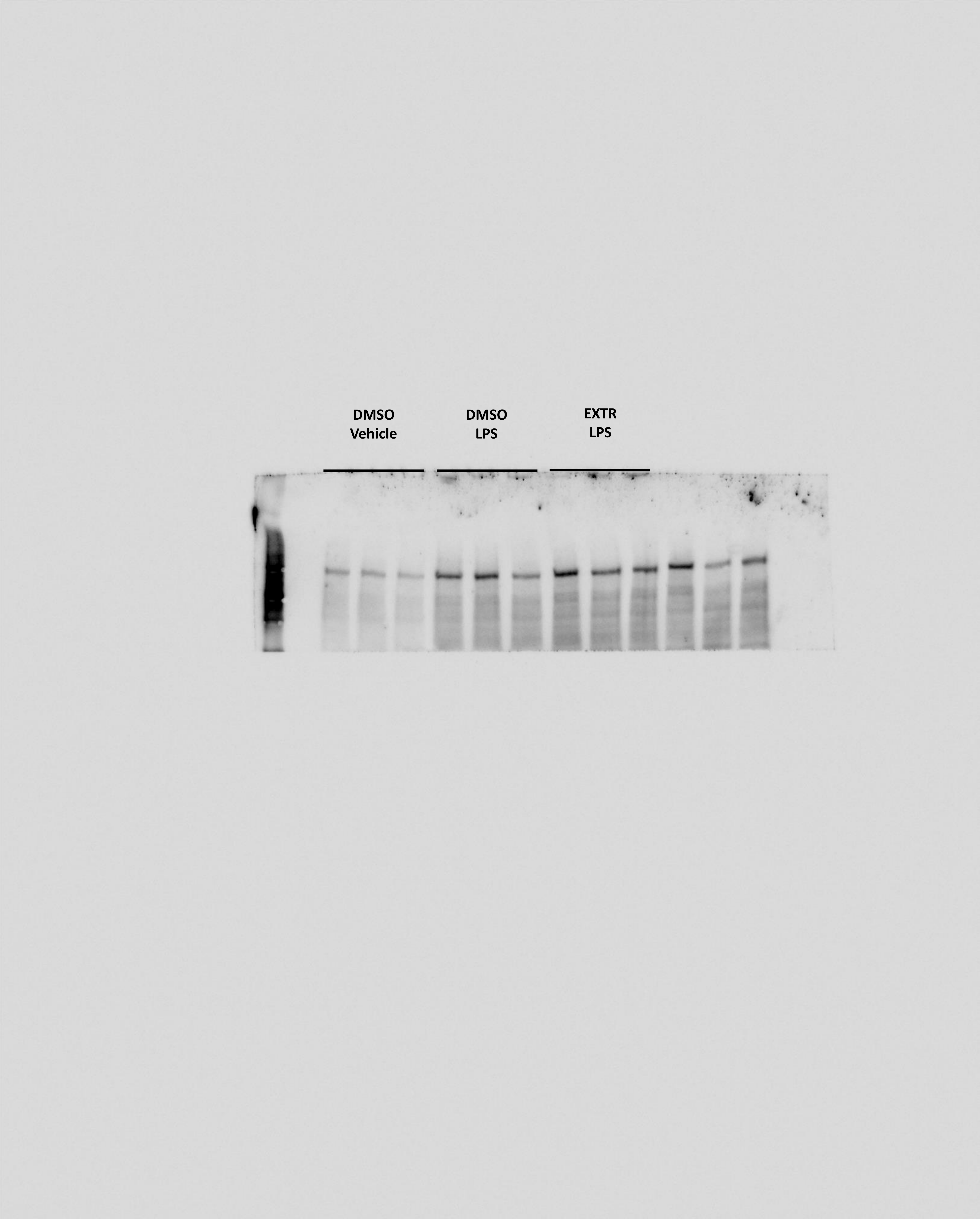

Figure 2A Total LRRK2 blot

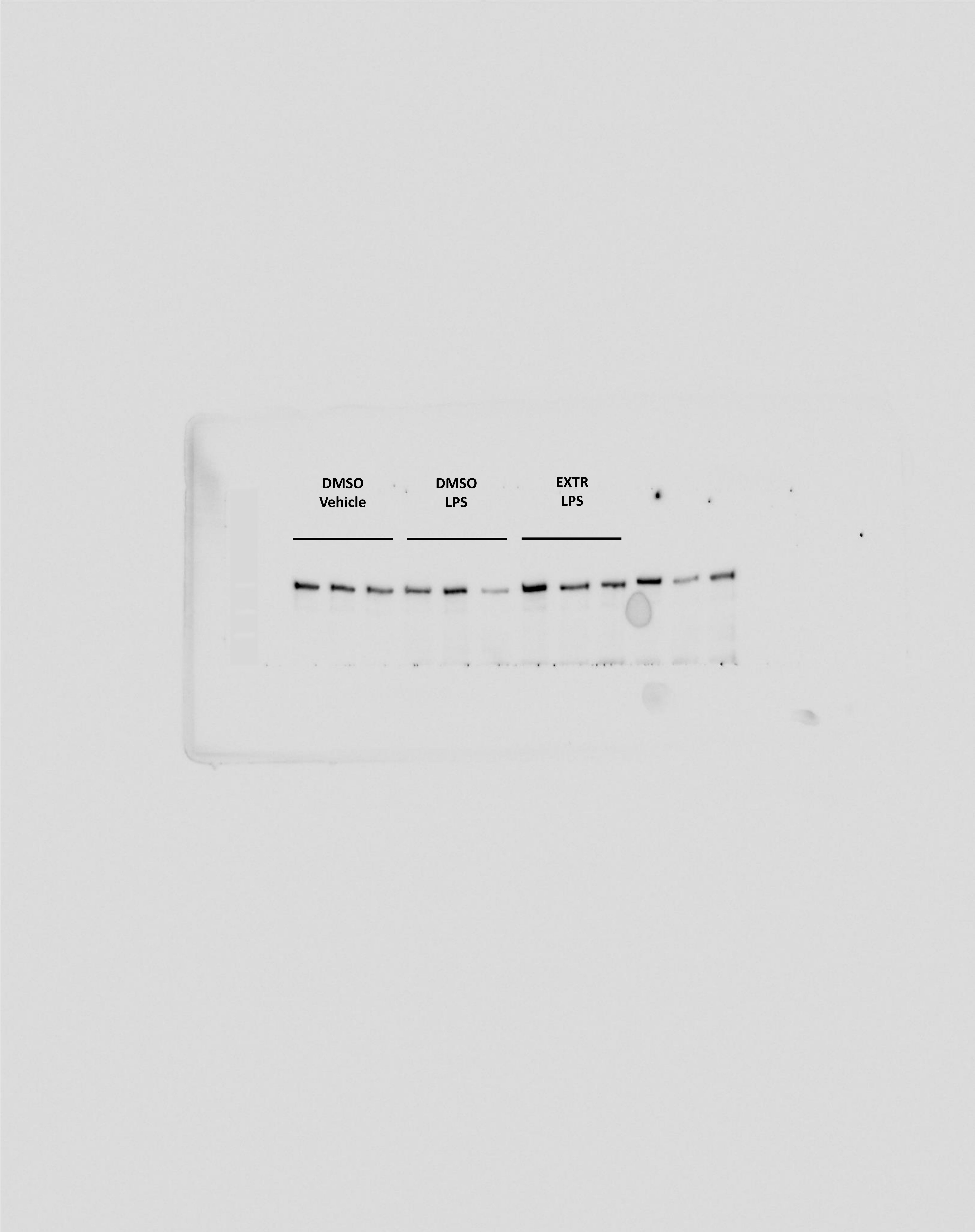

Figure 2A β-actin blot (5s)

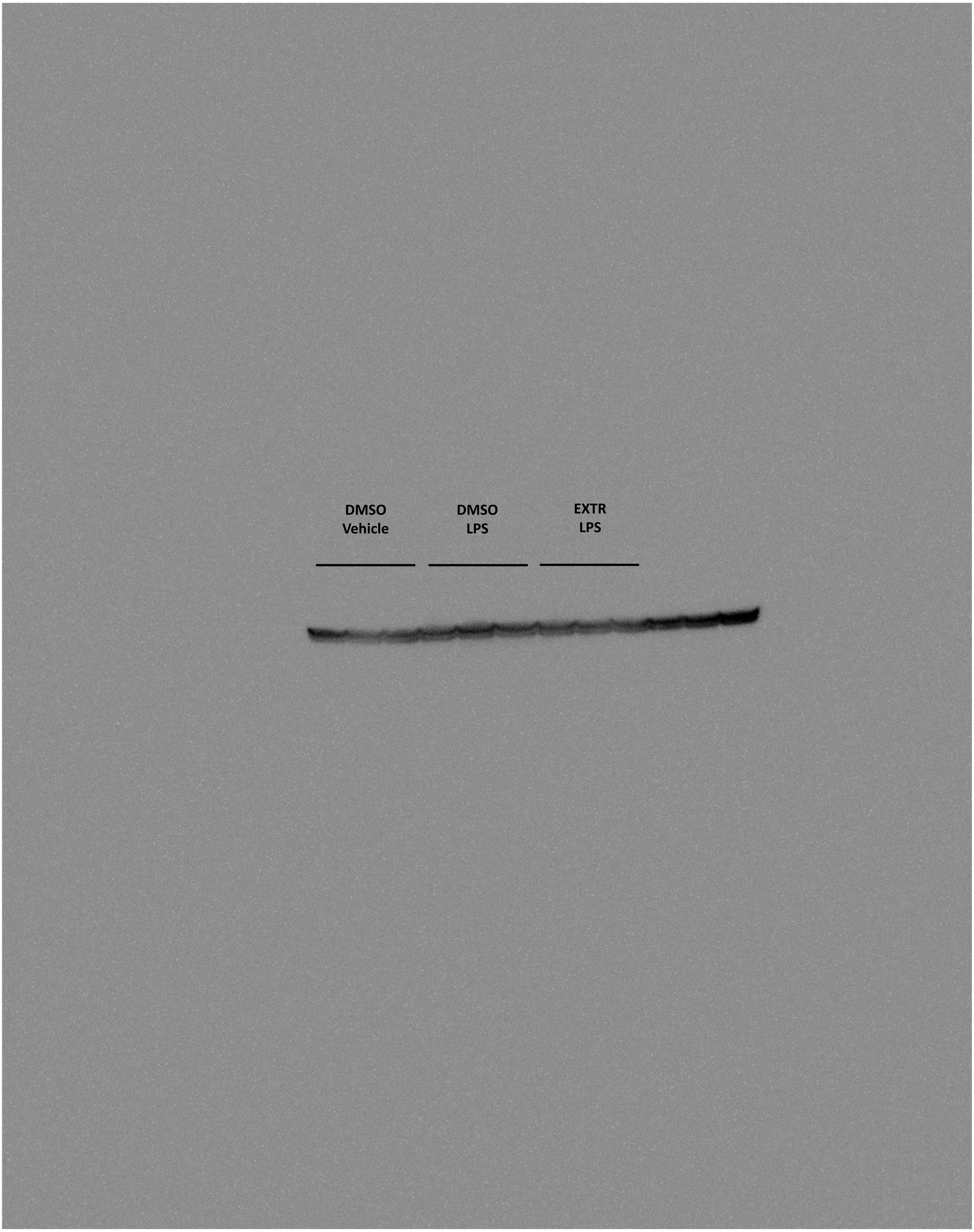

Figure 2A β-actin blot (0.5s)

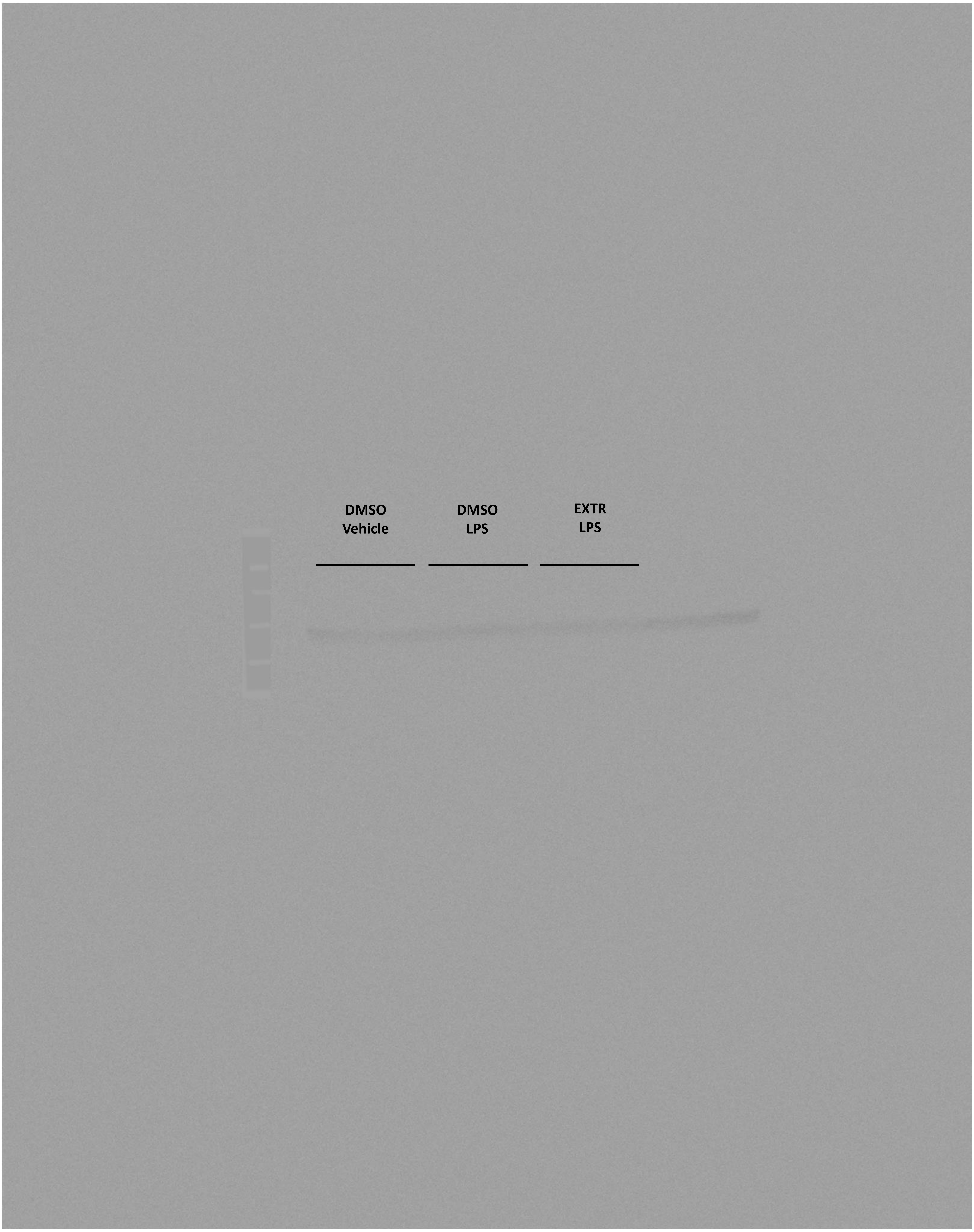

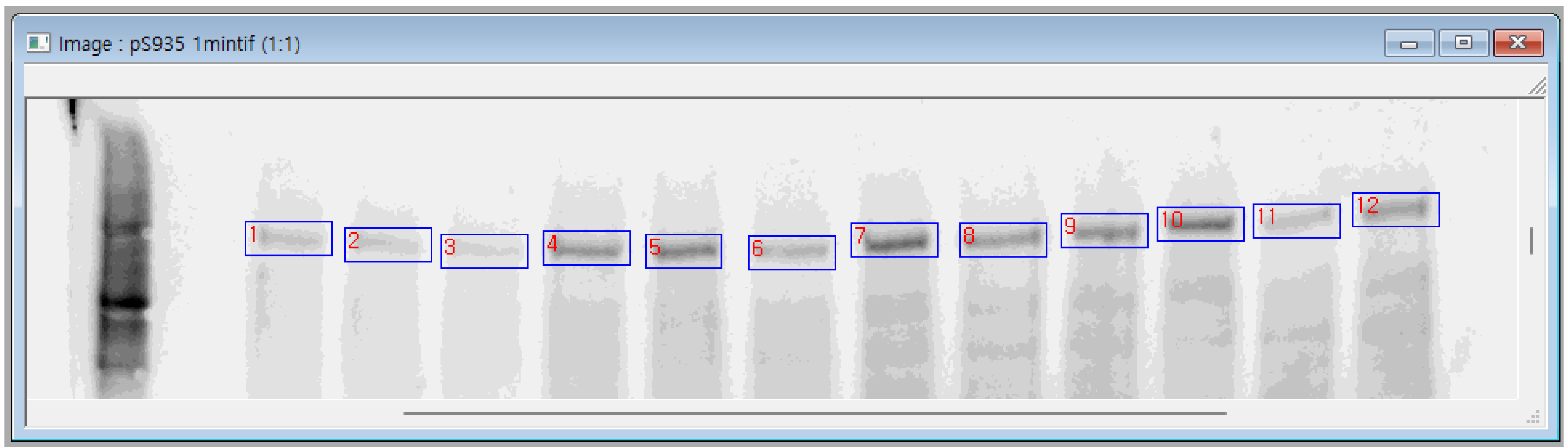

Result Table: pS935 1mintif [Quant] [Ratio(%): QL | Calibrated: ---]

Channel	No	Group	Index	Name	QL	Area(pixel2)	QL-BG	B	BG	Std	Ratio(%)	QL/pixel2	(Q-B)/pixel2	Calibrated
1	1	~	1		942540.00	1210.00	242196.00		700344.00		6.55	778.96	200.16	-------
1	2	~	2		870800.00	1210.00	170456.00		700344.00		6.05	719.67	140.87	-------
1	3	~	3		791613.00	1210.00	91269.00		700344.00		5.50	654.23	75.43	-------
1	4	~	4		1353489.00	1210.00	653145.00		700344.00		9.40	1118.59	539.79	-------
1	5	~	5		1340391.00	1056.00	729181.69		611209.31		9.31	1269.31	690.51	-------
1	6	~	6		1095625.00	1210.00	395281.00		700344.00		7.61	905.48	326.68	-------
1	7	~	7		1573390.00	1210.00	873046.00		700344.00		10.93	1300.32	721.53	-------
1	8	~	8		1350133.00	1210.00	649789.00		700344.00		9.38	1115.81	537.02	-------
1	9	~	9		1326051.00	1210.00	625707.00		700344.00		9.21	1095.91	517.11	-------
1	10	~	10		1501247.00	1210.00	800903.00		700344.00		10.43	1240.70	661.90	-------
1	11	~	11		973601.00	1210.00	273257.00		700344.00		6.76	804.63	225.83	-------
1	12	~	12		1278948.00	1210.00	578604.00		700344.00		8.88	1056.98	478.19	-------
1	13	~	13		700344.00	1210.00	0.00	✓ B	700344.00		-------	578.80	0.00	-------

All / Comparisons

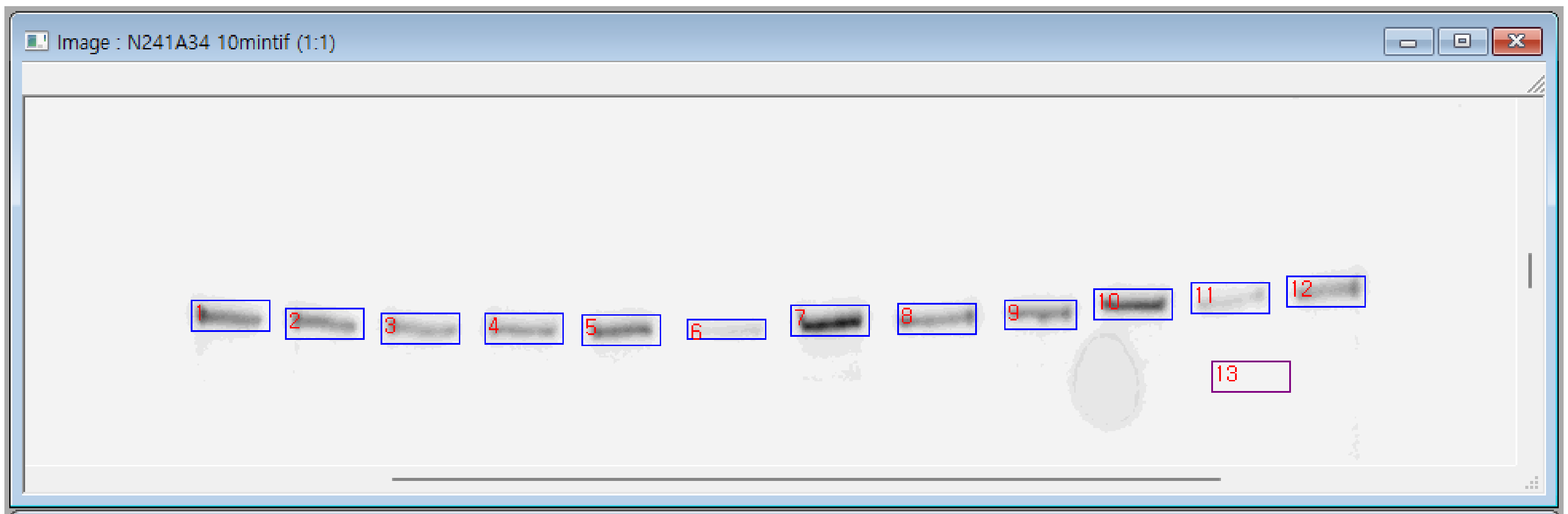

Result Table: N241A34 10mintif [Quant] [Ratio(%): QL | Calibrated: ---]

Channel	No	Group	Index	Name	QL	Area(pixel2)	QL-BG	B	BG	Std	Ratio(%)	QL/pixel2	(Q-B)/pixel2	Calibrated
1	1	~	1		1360696.00	1000.00	939425.00		421271.00		9.67	1360.70	939.42	-------
1	2	~	2		1188002.00	1000.00	766731.00		421271.00		8.44	1188.00	766.73	-------
1	3	~	3		1063573.00	1000.00	642302.00		421271.00		7.56	1063.57	642.30	-------
1	4	~	4		1075021.00	1000.00	653750.00		421271.00		7.64	1075.02	653.75	-------
1	5	~	5		1283679.00	1000.00	862408.00		421271.00		9.12	1283.68	862.41	-------
1	6	~	6		483365.00	650.00	209538.85		273826.15		3.43	743.64	322.37	-------
1	7	~	7		1925966.00	1000.00	1504695.00		421271.00		13.69	1925.97	1504.69	-------
1	8	~	8		1229248.00	1000.00	807977.00		421271.00		8.73	1229.25	807.98	-------
1	9	~	9		1100068.00	874.00	731877.15		368190.85		7.82	1258.66	837.39	-------
1	10	~	10		1516263.00	1000.00	1094992.00		421271.00		10.77	1516.26	1094.99	-------
1	11	~	11		726499.00	1000.00	305228.00		421271.00		5.16	726.50	305.23	-------
1	12	~	12		1120769.00	1000.00	699498.00		421271.00		7.96	1120.77	699.50	-------
1	13	~	13		421271.00	1000.00	0.00	✓ B	421271.00		-------	421.27	0.00	-------

All / Comparisons

	pS935	N241A	pS935/N241A	
DMSO	200.16	939.42	0.213068	1.243021
	140.87	766.73	0.183728	1.071858
	75.43	642.3	0.117437	0.685121
LPS	539.79	653.75	0.825683	4.816973
	690.51	862.41	0.800675	4.67108
	326.68	322.37	1.01337	5.911926
PA-EXT + LPS	721.53	1504.69	0.479521	2.797489
	537.02	807.98	0.664645	3.877492
	517.11	837.39	0.617526	3.602602

Figure 5A alpha-synuclein blot (5 min)

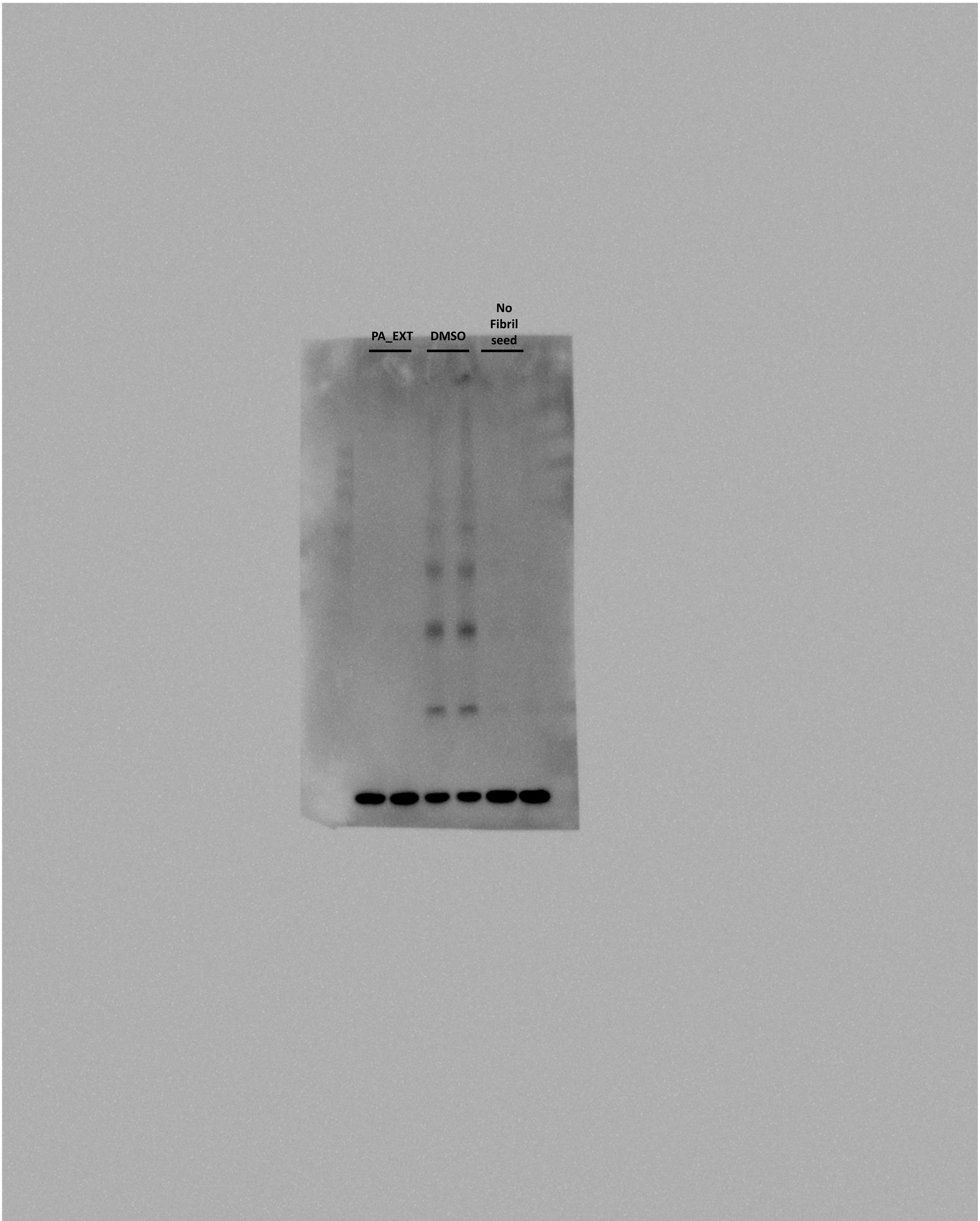

Figure 5A alpha-synuclein blot (1 min)

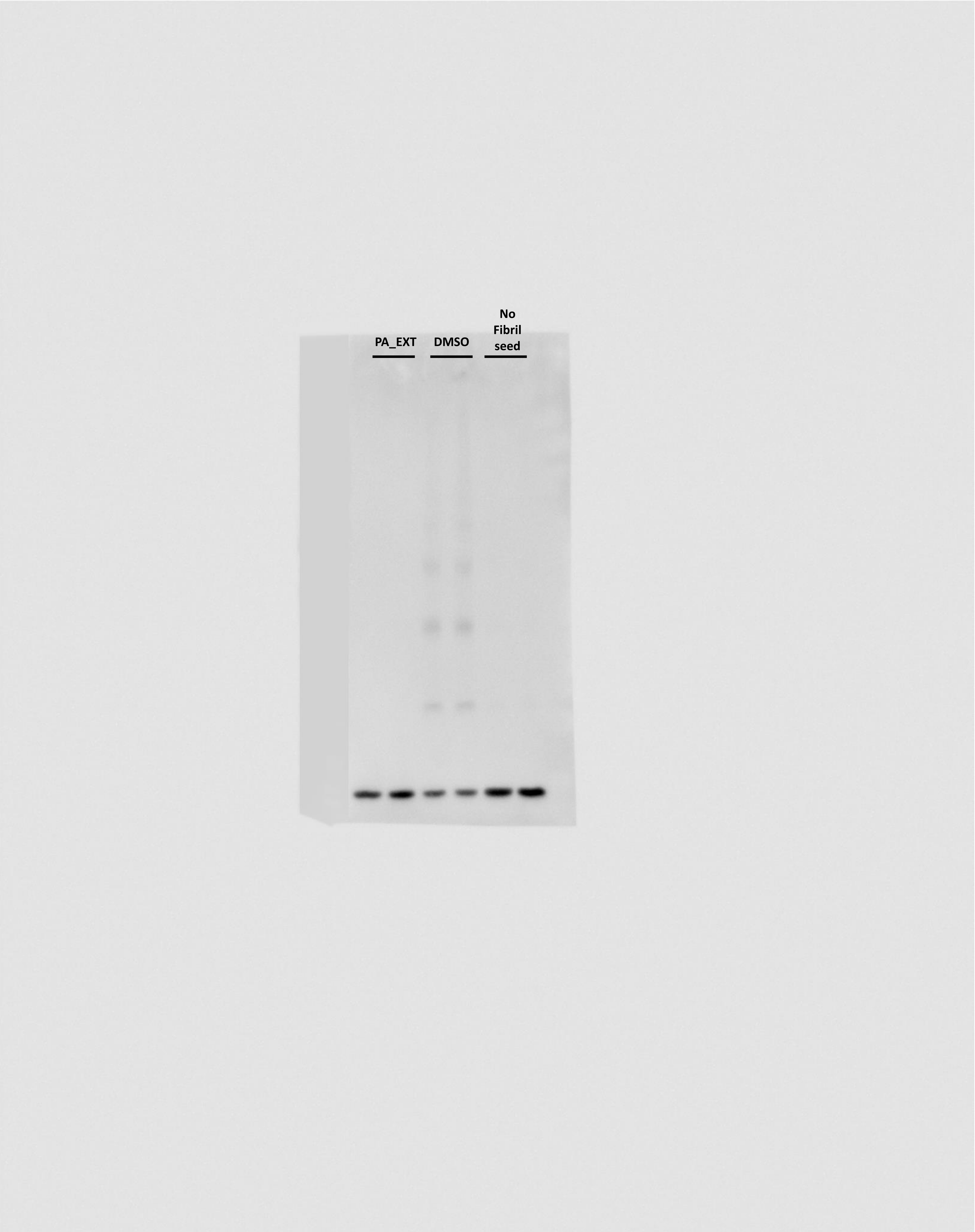

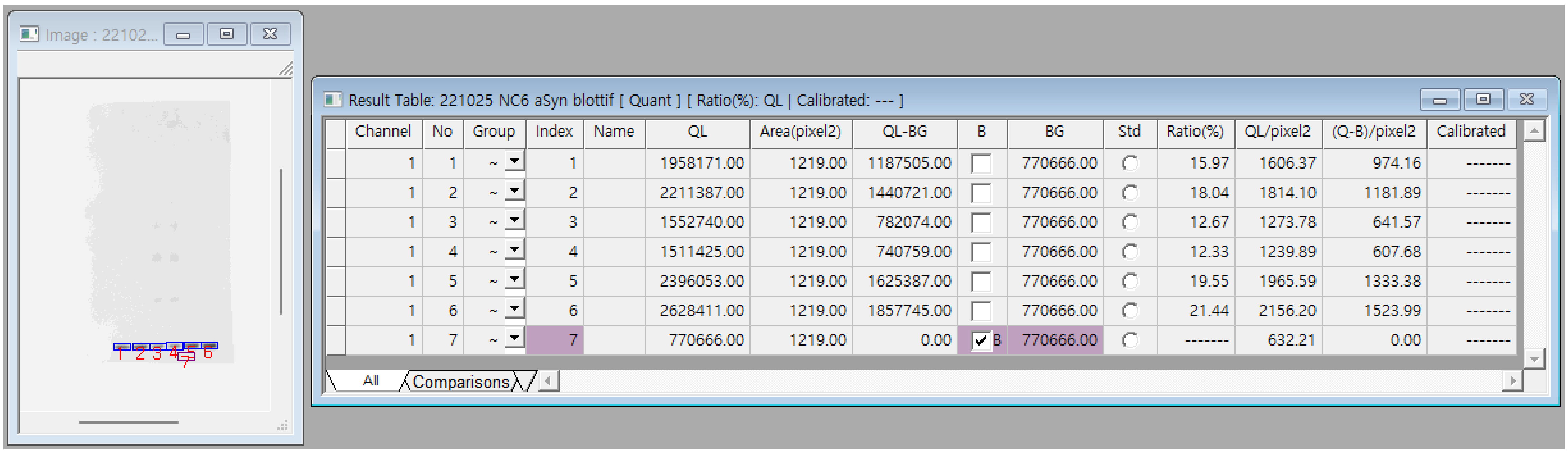
Image : 22102...

Result Table: 221025 NC6 aSyn blottif [Quant] [Ratio(%): QL | Calibrated: ---]

Channel	No	Group	Index	Name	QL	Area(pixel2)	QL-BG	B	BG	Std	Ratio(%)	QL/pixel2	(Q-B)/pixel2	Calibrated
1	1	~	1		1958171.00	1219.00	1187505.00		770666.00		15.97	1606.37	974.16	-------
1	2	~	2		2211387.00	1219.00	1440721.00		770666.00		18.04	1814.10	1181.89	-------
1	3	~	3		1552740.00	1219.00	782074.00		770666.00		12.67	1273.78	641.57	-------
1	4	~	4		1511425.00	1219.00	740759.00		770666.00		12.33	1239.89	607.68	-------
1	5	~	5		2396053.00	1219.00	1625387.00		770666.00		19.55	1965.59	1333.38	-------
1	6	~	6		2628411.00	1219.00	1857745.00		770666.00		21.44	2156.20	1523.99	-------
1	7	~	7		770666.00	1219.00	0.00	✓ B	770666.00		-------	632.21	0.00	-------

All | Comparisons

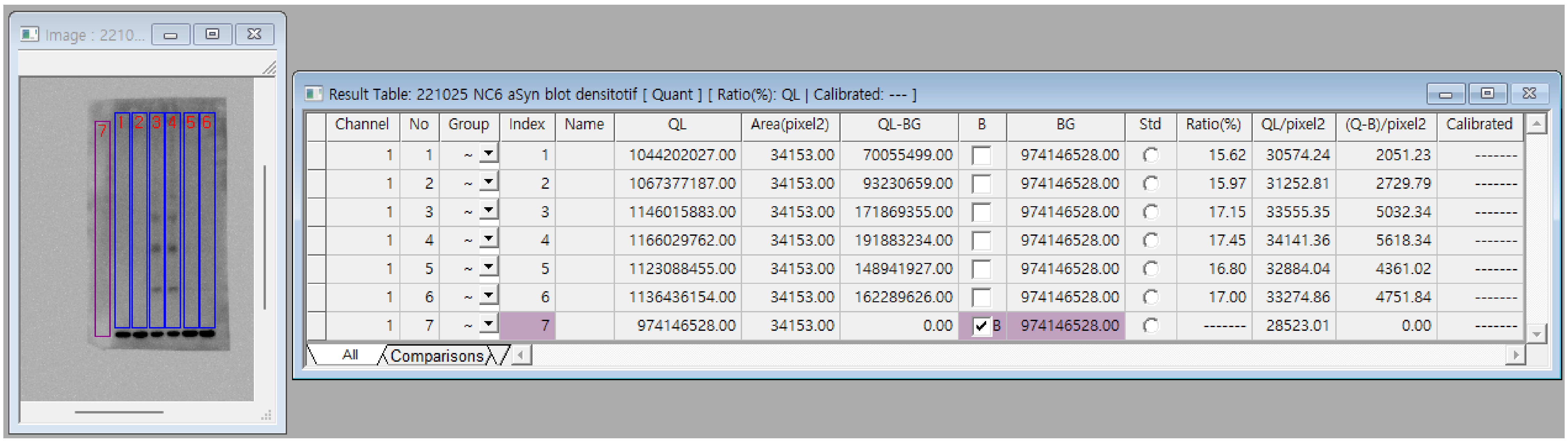
Image : 2210...

Result Table: 221025 NC6 aSyn blot densitotif [Quant] [Ratio(%): QL | Calibrated: ---]

Channel	No	Group	Index	Name	QL	Area(pixel2)	QL-BG	B	BG	Std	Ratio(%)	QL/pixel2	(Q-B)/pixel2	Calibrated
1	1	~	1		1044202027.00	34153.00	70055499.00		974146528.00		15.62	30574.24	2051.23	-------
1	2	~	2		1067377187.00	34153.00	93230659.00		974146528.00		15.97	31252.81	2729.79	-------
1	3	~	3		1146015883.00	34153.00	171869355.00		974146528.00		17.15	33555.35	5032.34	-------
1	4	~	4		1166029762.00	34153.00	191883234.00		974146528.00		17.45	34141.36	5618.34	-------
1	5	~	5		1123088455.00	34153.00	148941927.00		974146528.00		16.80	32884.04	4361.02	-------
1	6	~	6		1136436154.00	34153.00	162289626.00		974146528.00		17.00	33274.86	4751.84	-------
1	7	~	7		974146528.00	34153.00	0.00	✓ B	974146528.00		-------	28523.01	0.00	-------

All | Comparisons

	HMW	mono
PA_EXT	2051.23	974.16
	2729.79	1181.89
DMSO	5032.34	641.57
	5618.34	607.68
No Fibril seed	4361.02	1333.38
	4751.84	1523.99
